# Chemical Modification Strategies for Therapeutic Oligonucleotides: Mechanism Compatibility, Design Trade-Offs, and Translational Barriers

**DOI:** 10.3390/molecules31152588

**Published:** 2026-07-24

**Authors:** Kameron Burton, Kristen Dellinger

**Affiliations:** Department of Nanoengineering, Joint School of Nanoscience and Nanoengineering, North Carolina A&T State University, 2907 E Gate City Blvd, Greensboro, NC 27401, USA; kjburton@aggies.ncat.edu

**Keywords:** oligonucleotide therapeutics, chemical modification, antisense oligonucleotides, small interfering RNA, nuclease resistance, drug delivery, nucleic acid chemistry, medicinal chemistry

## Abstract

Oligonucleotide therapeutics represent an expanding class of medicines that can regulate gene expression, RNA processing, protein translation, immune signaling, and biomolecular recognition through sequence-specific or structure-dependent mechanisms. Despite clinical progress, their application remains constrained by nuclease degradation, rapid clearance, inefficient tissue and cellular delivery, endosomal sequestration, off-target activity, immune recognition, and mechanism-specific requirements for target engagement. Chemical modification is central to oligonucleotide therapeutic development because it can mitigate some of these limitations while influencing target affinity, protein binding, pharmacokinetics, and intracellular activity. This review examines chemical modification strategies to improve the biological stability and functional performance of therapeutic oligonucleotides and is organized around major classes of chemical modification, including phosphate and backbone-linkage modifications, sugar and conformational modifications, backbone-replacement analogs, and conjugation-based approaches. Rather than presenting these chemistries as uniformly beneficial, this review emphasizes that the same modification can be enabling in one therapeutic mechanism and disruptive in another, so its value cannot be judged apart from the modality and molecular architecture in which it is placed. Clinically successful oligonucleotide designs are likely to rely on combinations of chemical features, including modified backbones, modified sugars, stereochemical control, terminal stabilization, and ligand- or formulation-based delivery strategies. Understanding how these features interact is essential to develop more predictable and mechanism-appropriate oligonucleotide therapeutics.

## 1. Introduction

Oligonucleotides are short single-stranded nucleic acids, typically composed of approximately 13–25 nucleotides, that can be synthesized with high sequence precision and tailored to hybridize with complementary DNA or RNA targets [1,2]. Since the early development of synthetic oligonucleotide chemistry, these molecules have become valuable tools across molecular biology, biotechnology, and medicine due to their programmability, relative ease of synthesis, and capacity for sequence-specific recognition. Their ability to engage nucleic acid targets through predictable base-pairing interactions has enabled broad utility in nucleic acid detection, amplification, sequencing, and gene regulation. More importantly, these same molecular properties have positioned oligonucleotides as a compelling therapeutic platform for modulating disease-associated pathways at the RNA or DNA level.

The therapeutic significance of oligonucleotides lies in their capacity to intervene upstream of protein expression and function. Unlike conventional small molecules, which typically act by binding to proteins, oligonucleotide therapeutics can be designed to selectively recognize nucleic acid sequences and influence transcriptional or post-transcriptional events with high specificity. This has led to the development of multiple oligonucleotide-based modalities, including antisense oligonucleotides (ASOs), small interfering RNAs (siRNAs), microRNA-modulating oligonucleotides, aptamers, decoy oligonucleotides, immunostimulatory oligonucleotides, and catalytic nucleic acids such as ribozymes and DNAzymes [3]. Although these platforms differ in structure and mechanism, they are unified by a central principle: sequence-directed recognition can be leveraged to regulate biological function in a programmable manner. This feature has made oligonucleotide therapeutics particularly attractive for diseases that are difficult to address through traditional pharmacological approaches, including those involving aberrant splicing, toxic RNA transcripts, dysregulated gene expression, or undruggable intracellular targets.

Despite this promise, the translation of oligonucleotides into effective therapeutic agents has been constrained by substantial biological and physicochemical barriers. Native DNA and RNA are not intrinsically optimized for persistence or function in vivo. Under physiological conditions, oligonucleotides are susceptible to chemical and enzymatic degradation, including nuclease-mediated cleavage of phosphodiester linkages, oxidative damage, and other forms of molecular instability [4,5,6]. In the bloodstream, plasma protein binding governs how long an oligonucleotide persists and where it distributes, and this behavior is set largely by backbone chemistry rather than by the strand itself: substitution of a non-bridging oxygen with sulfur increases both plasma stability and plasma protein binding, which limits glomerular filtration and urinary excretion and thereby raises tissue bioavailability [7,8]. This distinction matters when interpreting the delivery literature, much of which characterizes formulated particles such as lipoplexes and polyplexes, whose distribution and cellular entry are governed by the carrier rather than by the chemistry of the oligonucleotide it carries [9,10,11]. At the cellular level, oligonucleotides enter by endocytosis and must then escape the endosome, a step so inefficient that only a small fraction of internalized material, on the order of one to two percent, reaches the cytosol or nucleus; the remainder is degraded in lysosomes or recycled back out of the cell [12,13]. Productive release occurs principally from late endosomes and depends on host trafficking machinery rather than on the oligonucleotide alone [14]. For oligonucleotides intended to act within the nucleus, an additional barrier is imposed by nuclear membrane transport, which can further limit target engagement [15]. Collectively, these challenges reduce bioavailability, compromise pharmacokinetics, and shorten the effective half-life of unmodified oligonucleotides, thereby limiting their therapeutic performance.

These limitations have driven the extensive chemical engineering of oligonucleotide scaffolds to improve their biological stability, functional activity, and delivery characteristics. Rather than relying on native nucleic acid chemistry, therapeutic oligonucleotides are frequently modified at the phosphate backbone, sugar moiety, nucleobases, or termini, or conjugated with functional ligands and delivery-promoting groups [16,17,18,19]. Such modifications rarely act on a single property: one chemical change can simultaneously shift nuclease resistance, target affinity, protein binding, immune recognition, and biodistribution, which is why their effects must be weighed against one another rather than simply added up [20,21,22,23,24,25]. Importantly, the benefits of chemical modification are not universally equivalent across all therapeutic contexts. A modification that is advantageous for one oligonucleotide class may be less suitable for another depending on the intended mechanism of action, required intracellular destination, protein interactions, or safety profile. Thus, the design of oligonucleotide therapeutics is not simply a matter of increasing stability, but of balancing stability with functional compatibility, specificity, delivery efficiency, and tolerability.

As the field has matured, it has become increasingly clear that chemical modification is not a secondary optimization step, but a foundational determinant of therapeutic efficacy. However, the effects of a given modification cannot be interpreted independently of oligonucleotide architecture, target site, intracellular destination, and mechanism of action. Chemistries that improve nuclease resistance or target affinity may also alter protein binding, biodistribution, immune recognition, enzymatic compatibility, or intracellular activity. Accordingly, therapeutic oligonucleotide design is best understood as a balance of stability, specificity, delivery, safety, and functional compatibility rather than maximizing any single property.

In this review, we examine chemical modification strategies to improve the biological stability and functional performance of therapeutic oligonucleotides, emphasizing how individual modifications have been matched to therapeutic mechanisms. The discussion is organized around phosphate and backbone-linkage modifications, sugar and conformational modifications, backbone-replacement analogs, and conjugation-based approaches. While focus is placed on how these strategies influence nuclease resistance, target affinity, pharmacokinetic behavior, enzyme compatibility, delivery, and safety. Context-dependent trade-offs and the increasing importance of combined chemical architectures in clinically successful and translationally advanced oligonucleotide designs is also discussed.

## 2. Oligonucleotide Therapeutic Classes and Shared Biological Barriers

### 2.1. Major Therapeutic Classes

**Figure 1:**
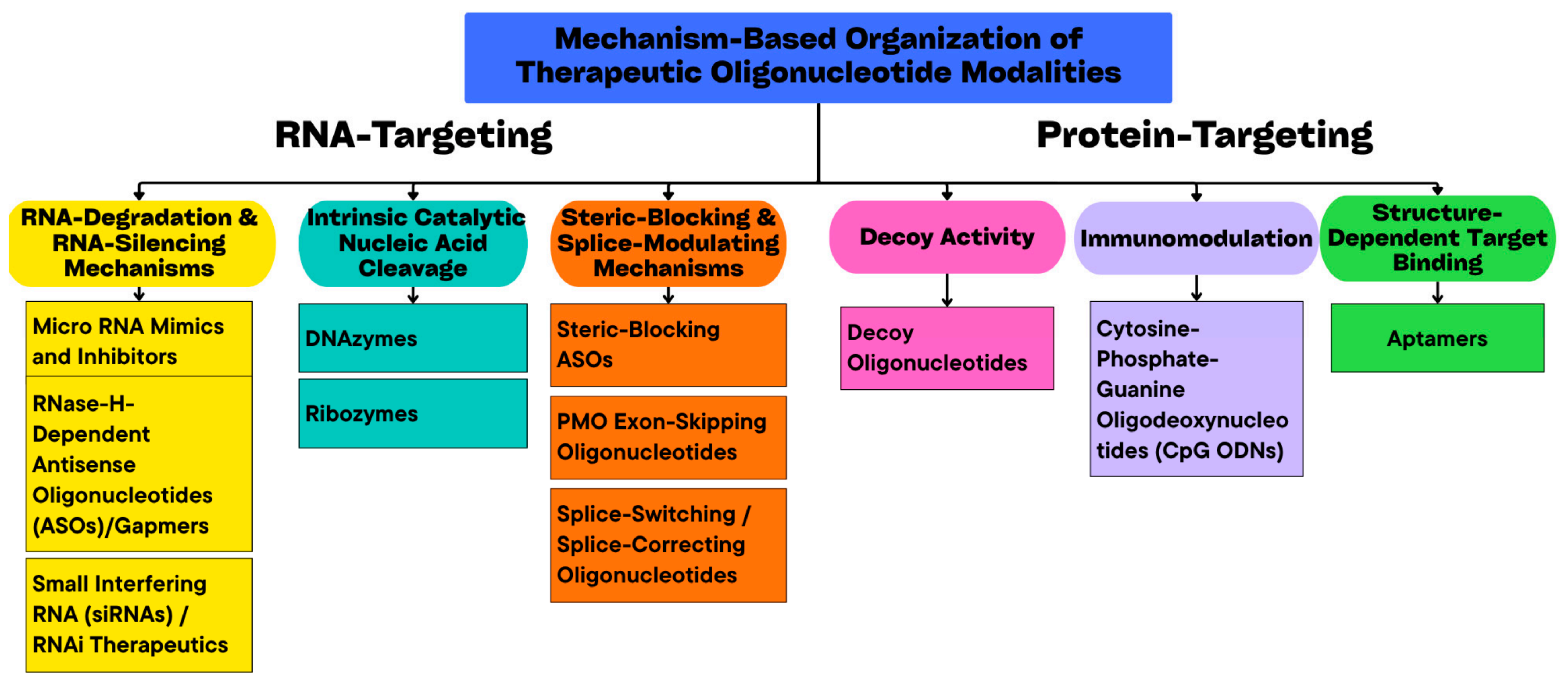
Mechanism-based classification of therapeutic oligonucleotide strategies.

Oligonucleotide therapeutics are a diverse group of synthetic nucleic acid-based agents designed to modulate gene expression, RNA processing, protein translation, immune signaling, or biomolecular recognition through sequence-specific or structure-dependent interactions. Although these modalities differ in chemical architecture, intracellular site of action, and mechanism of activity, they share a common therapeutic premise: nucleic acid sequence recognition or nucleic acid folding can be used to regulate biological function with high programmability and target selectivity. Here, the major therapeutic classes are organized according to their primary mechanism of action, including ribonuclease H (RNase H)-dependent antisense oligonucleotides (ASOs)/gapmers, steric-blocking ASOs, splice-switching or splice-correcting oligonucleotides, small interfering RNAs (siRNAs)/RNA interference (RNAi) therapeutics, microRNA (miRNA) mimics and inhibitors, aptamers, cytosine–phosphate–guanine oligodeoxynucleotides (CpG ODNs)/immunostimulatory oligonucleotides, decoy oligonucleotides, and catalytic nucleic acids such as DNAzymes and ribozymes (Figure 1). These distinctions are important because chemical modifications do not affect every oligonucleotide modality in the same way [26,27,28,29]. For example, RNase H-dependent ASOs must preserve an architecture compatible with RNase H-mediated cleavage [30,31], whereas splice-switching oligonucleotides rely on sustained binding to accessible splice-regulatory regions without intentionally promoting RNA degradation [32,33]. In siRNA therapeutics, modification patterns must remain compatible with RNAi pathway loading and guide-strand function [34,35,36]; in aptamers and catalytic nucleic acids, chemical changes must preserve folded binding structures [37,38] or catalytic activity [39,40,41].

Therapeutic oligonucleotides can be organized according to the primary biological mechanism through which they alter molecular function. RNase H-dependent ASOs/gapmers, siRNAs, and miRNA mimics or inhibitors are grouped together because they regulate target RNA abundance or RNA-mediated gene regulation through RNA degradation, RNA interference, or miRNA pathway modulation. Steric-blocking and splice-switching oligonucleotides, including PMO exon-skipping designs, act primarily by occupying RNA sites to alter splicing, translation, or RNA–protein interactions without intentionally recruiting target RNA cleavage. DNAzymes and ribozymes are grouped as catalytic nucleic acids because their activity depends on sequence-guided positioning of catalytic motifs for RNA cleavage or RNA-processing reactions. Aptamers function through structure-dependent target binding, CpG oligodeoxynucleotides act through innate immune stimulation or immunomodulation, and decoy oligonucleotides mimic regulatory DNA elements to sequester transcription factors.

**Figure 2:**
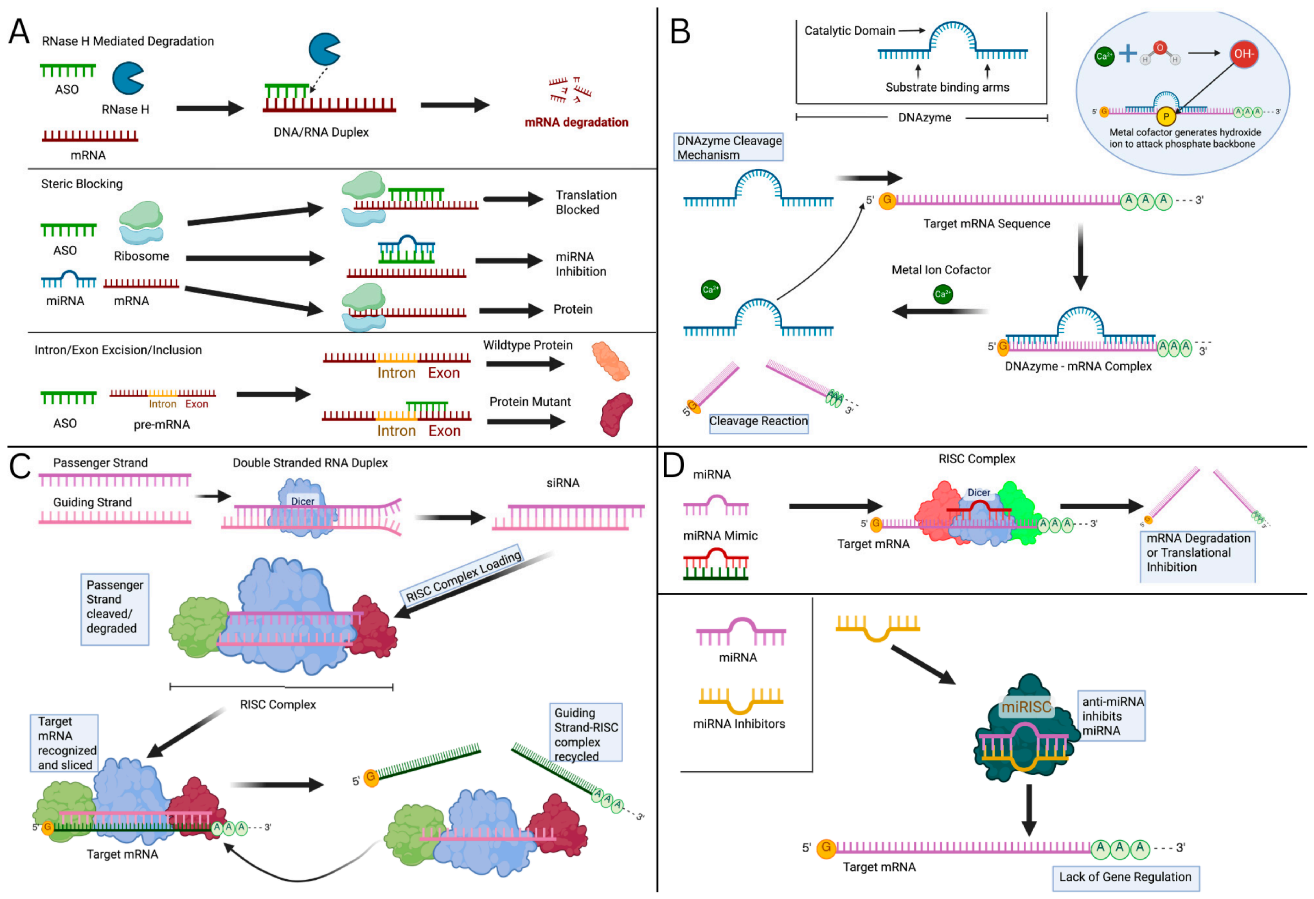
Representative gene-regulatory oligonucleotide mechanisms. (**A**) Antisense oligonucleotide (ASO) mechanisms. RNase H-dependent ASOs hybridize with complementary RNA sequences to form RNA/DNA duplexes that support RNase H-mediated cleavage of the RNA strand, reducing target transcript abundance. Steric-blocking ASOs regulate gene expression without intentionally promoting target RNA cleavage by occupying RNA regions involved in translation, RNA–protein interactions, or other regulatory processes. Splice-switching ASOs bind pre-mRNA at splice-regulatory regions to alter exon or intron recognition, thereby changing splicing outcomes and protein isoform expression. (**B**) DNAzyme-mediated RNA cleavage. RNA-cleaving DNAzymes contain a catalytic domain flanked by substrate-binding arms that hybridize to a target RNA sequence. Upon formation of the DNAzyme–RNA complex, the catalytic core promotes site-specific cleavage of the target RNA, often with the assistance of divalent metal ions depending on the DNAzyme system. Following cleavage, product fragments can dissociate, allowing the DNAzyme to participate in additional catalytic cycles. (**C**) Small interfering RNA (siRNA) pathway. A double-stranded siRNA duplex composed of guide and passenger strands is loaded into the RNA-induced silencing complex (RISC), where the guide strand directs sequence-specific recognition of complementary target transcripts. Productive guide-strand loading enables Argonaute-associated silencing, which can include endonucleolytic slicing of highly complementary target RNA. (**D**) microRNA (miRNA)-based therapeutic modulation. miRNA mimics are designed to restore or enhance miRNA-mediated regulation by entering the cellular silencing machinery and directing repression or destabilization of target transcripts. Anti-miRNA oligonucleotides bind endogenous miRNAs and reduce their ability to associate productively with target transcripts, thereby altering downstream miRNA-mediated gene regulation. Created in BioRender. Dellinger, K. (2026) (**A**) https://BioRender.com/gdndn8a (**B**) https://BioRender.com/9cvfilb (**C**) https://BioRender.com/htzjfvl (**D**) https://BioRender.com/hd4tjri. All accessed on 31 May 2026.

Antisense oligonucleotides are typically short single-stranded oligonucleotides that bind complementary messenger RNA (mRNA) or pre-mRNA sequences through Watson–Crick base pairing to alter gene expression. Their mechanisms include RNase H-mediated degradation of target RNA [42,43,44], steric blocking of translation or RNA–protein interactions [45,46,47], and modulation of splice-site selection [48,49,50] (Figure 2). ASO performance depends not only on chemical stabilization but also on target-site selection [51,52], RNA accessibility [53,54], sequence discrimination [55,56], and compatibility between modification pattern and intended mechanism.

Small interfering RNAs are double-stranded RNA molecules that act through the RNAi pathway to reduce target RNA expression. After intracellular delivery, the guide strand is loaded into the RNA-induced silencing complex (RISC), where Argonaute-containing complexes use sequence complementarity to recognize target transcripts and promote silencing [57,58]. The therapeutic performance of siRNAs depends on more than duplex stability alone; chemical modification must preserve productive RISC loading, guide-strand activity, and appropriate strand bias while reducing nuclease susceptibility, immune activation, and off-target effects. Because partial complementarity, especially through seed-region interactions [59,60], can contribute to unintended transcript regulation [61,62,63], siRNA design requires coordination between sequence selection, chemical modification patterning, and delivery strategy.

miRNA-based therapeutics include miRNA mimics and miRNA inhibitors. miRNA mimics are commonly designed to restore or enhance the activity of miRNA pathways that are reduced in disease, whereas miRNA inhibitors, including antagomirs or anti-miRs, bind endogenous miRNAs and reduce their ability to regulate target transcripts [64,65]. Unlike siRNAs, which are typically designed for high complementarity to a target transcript, endogenous miRNAs often regulate gene expression through partial complementarity, frequently involving sites in the 3′ untranslated region (3′ UTR) of target mRNAs [66,67]. This mode of regulation allows a single miRNA to influence multiple transcripts, which can be therapeutically useful when a disease-relevant pathway is dysregulated but also may increase the risk of broad transcriptome-level effects or unintended pathway perturbation [68,69]. For this reason, miRNA therapeutic design must account not only for stability and delivery but also for target-network specificity and potential interference with endogenous RNAi-associated regulatory pathways.

Aptamers are single-stranded DNA or RNA oligonucleotides that are commonly identified through systematic evolution of ligands by exponential enrichment (SELEX), an iterative selection process that enriches high-affinity binders from randomized oligonucleotide libraries. Their function depends on their ability to fold into defined secondary or tertiary structures capable of recognizing proteins, small molecules, or cellular targets [70]. Unlike hybridization-dependent modalities, aptamers rely primarily on structure-dependent molecular recognition, making their activity closely tied to preservation of conformational integrity and binding-site geometry [71]. Therapeutic aptamers may function through direct target inhibition [72], receptor modulation [73], immune pathway regulation [74], or as targeting ligands for delivery systems [75], depending on the selected target and structural design. This mechanism places different chemical constraints on aptamers than on RNA-degrading or steric-blocking oligonucleotides: modifications must improve stability and persistence without disrupting the folded structure required for target binding.

Ribozymes are catalytic RNA molecules that can promote site-specific RNA cleavage, ligation, or splicing-related reactions depending on ribozyme class and biological context [76,77]. Their therapeutic relevance arises from the ability to combine sequence-guided target recognition with catalytic activity, most commonly in strategies intended to alter RNA-level gene expression or RNA processing [78]. Because ribozyme function depends on higher-order folding and positioning of catalytic groups [79], chemical modification must be evaluated carefully to avoid disrupting the structure required for substrate recognition and catalysis. This distinguishes ribozymes from simpler hybridization-dependent oligonucleotides: increasing nuclease resistance or binding affinity may be beneficial only if catalytic folding, intracellular stability, and target-site accessibility are preserved.

Decoy oligonucleotides are typically short double-stranded DNA constructs designed to mimic transcription factor binding sites and competitively sequester transcription factors away from endogenous regulatory elements. Unlike hybridization-dependent modalities that act through complementary RNA recognition, decoy oligonucleotides function through protein–DNA recognition and depend on the ability of the decoy sequence to reproduce the relevant transcription factor binding motif [80,81]. Their activity therefore depends on stability, intracellular and nuclear availability, sufficient local concentration to compete with genomic binding sites, and preservation of transcription factor binding specificity [82,83]. Chemical modification of decoy oligonucleotides must be evaluated in this context because changes that improve nuclease resistance or persistence may also alter protein-binding affinity, specificity, or cellular distribution.

**Figure 3:**
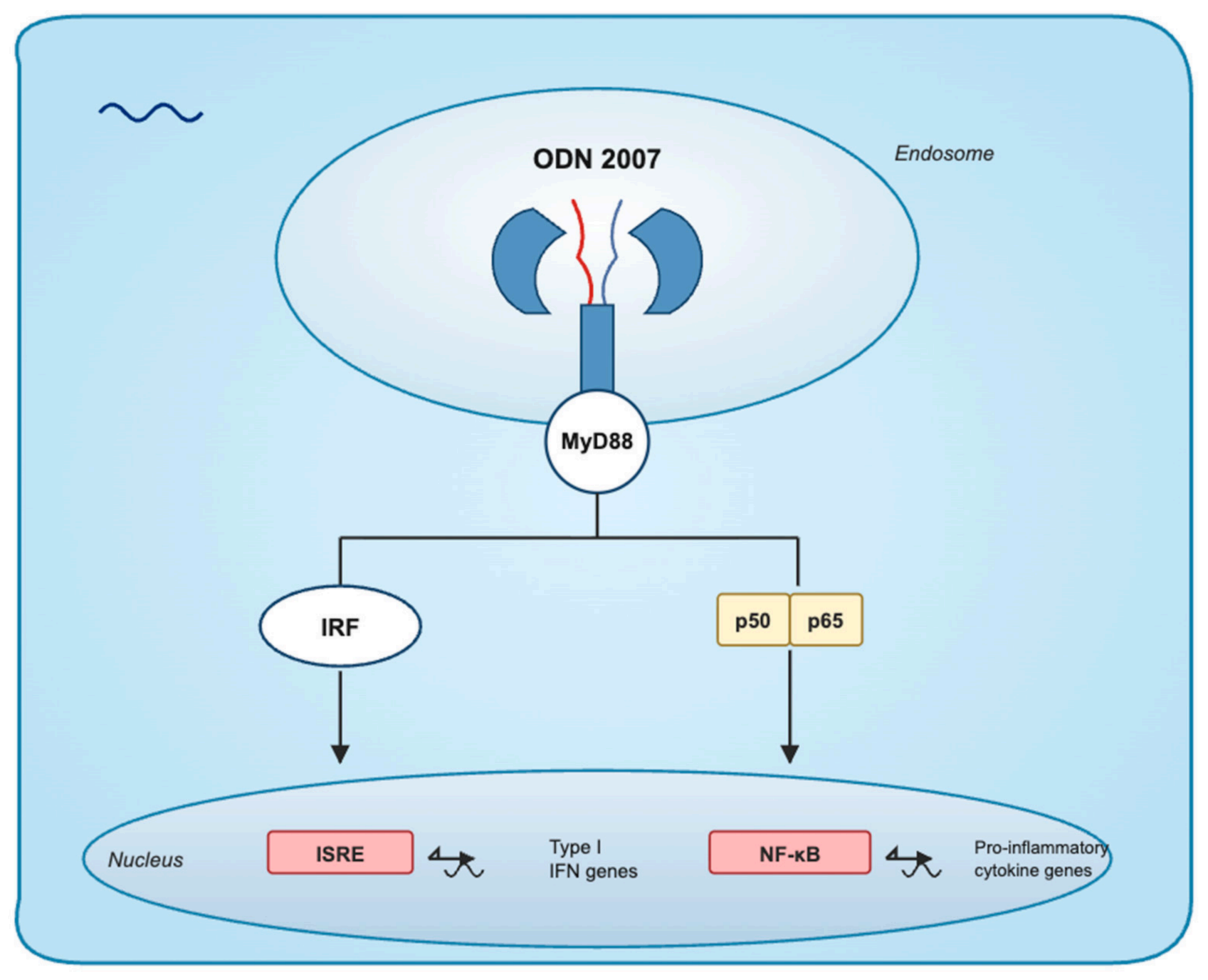
Immunomodulatory oligonucleotide mechanism. Following endosomal uptake, CpG oligodeoxynucleotides engage MyD88-associated signaling and regulate downstream IRF- and NF-κB-related transcriptional responses involved in immune activation.

CpG oligodeoxynucleotides are synthetic DNA sequences containing unmethylated cytosine–phosphate–guanine motifs that can stimulate innate immune signaling through Toll-like receptor 9 (TLR9) [84]. This modality is mechanistically distinct from RNA-silencing or splice-modulating oligonucleotides because immune activation is an intended therapeutic function rather than an adverse effect to be avoided. CpG ODNs have been grouped into classes with different sequence features, backbone compositions, structural properties, and immunostimulatory profiles, which can influence activation of plasmacytoid dendritic cells, B cells, interferon-associated responses, or antibody-promoting immune activity (Figure 3; the class B (Type K) CpG ODN 2007 is shown as a representative structure). Their therapeutic relevance is particularly important in vaccine adjuvant design and cancer immunotherapy, where controlled innate immune activation may support antigen-specific or antitumor responses [85,86]. For this reason, chemical modification of CpG ODNs should balance nuclease resistance and biological persistence with maintenance of TLR9 engagement and avoidance of excessive inflammatory activation [87,88].

DNAzymes, or deoxyribozymes, are catalytic DNA molecules that contain substrate-recognition regions and a catalytic core capable of promoting sequence-guided chemical reactions, including RNA cleavage and, in some systems, DNA cleavage or other nucleic acid transformations. In therapeutic gene-regulation contexts, DNAzymes have most often been investigated as RNA-targeting catalytic oligonucleotides that bind complementary transcript regions and promote site-specific cleavage [89,90,91]. Their potential advantage lies in combining sequence specificity with catalytic turnover, allowing a single DNAzyme molecule to participate in multiple cleavage events if substrate binding, catalysis, and product release are preserved. However, DNAzyme performance depends strongly on target-site accessibility; catalytic-core integrity; flanking-arm design; intracellular delivery; and, for many RNA-cleaving DNAzymes, availability of suitable metal ion cofactors. Chemical modification must therefore be positioned carefully to improve stability without disrupting substrate recognition, catalytic folding, or turnover [92].

### 2.2. Common Biological and Pharmacological Barriers

Although the therapeutic modalities described above differ substantially in mechanism, their development is shaped by recurring barriers, including nuclease degradation, rapid clearance, inefficient delivery, intracellular trafficking limitations, off-target activity, and immune recognition [93,94,95]. These barriers are shared across many oligonucleotide classes and should not be interpreted as belonging uniquely to any single modality. Instead, their relative importance depends on where the oligonucleotide must act, what molecular machinery it must engage or avoid, and which structural or catalytic features must be preserved for function. For example, a modification that improves nuclease resistance may benefit one modality but interfere with RNase H recruitment, RISC loading, splice modulation, aptamer folding, or catalytic turnover in another.

This mechanism-dependent balance provides the rationale for evaluating oligonucleotide chemistry beyond simple stabilization. Chemical modifications must be considered in relation to target-site accessibility, intracellular localization, protein or enzyme engagement, immune recognition, and the intended mode of action. Table 1 therefore summarizes the major therapeutic modalities according to their primary mechanisms and mechanism-specific design constraints, providing a framework for the backbone, sugar, conformational, and related modification strategies discussed in the sections that follow.

## 3. Phosphate-Linkage and Backbone-Replacement Strategies

**Figure 4:**
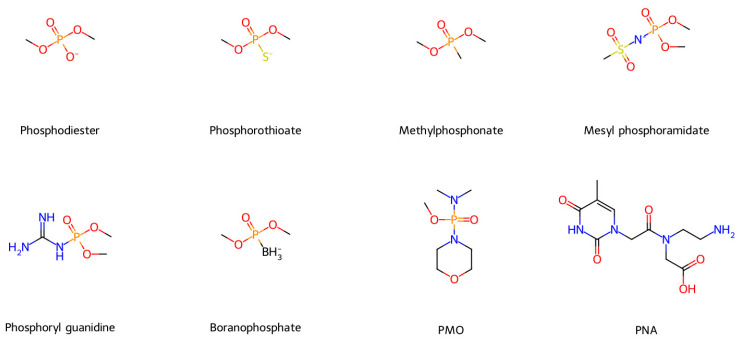
Phosphate-linkage and backbone-replacement chemistries for therapeutic oligonucleotides, organized relative to the native phosphodiester linkage. Top and middle rows: phosphate-linkage modifications that retain the native sugar–phosphate framework and differ only by replacement of a non-bridging phosphate oxygen. The native phosphodiester (boxed) is shown as the reference and is anionic. In the phosphorothioate (PS) linkage, a non-bridging oxygen is replaced by sulfur, producing an anionic backbone with a stereogenic phosphorus (R_p_/S_p_) that improves nuclease resistance and protein binding while remaining compatible with RNase H in DNA gap regions. Phosphorodithioate (PS_2_) replaces both non-bridging oxygens with sulfur and is therefore anionic but achiral. Methylphosphonate (alkylphosphonate) replaces a non-bridging oxygen with an alkyl group, giving a charge-neutral, stereogenic linkage. Boranophosphate replaces a non-bridging oxygen with a borane (BH_3_) group, retaining an anionic, stereogenic backbone. Mesyl phosphoramidate (MsPA) replaces a non-bridging oxygen with an N-methanesulfonyl amido group, yielding an anionic, stereogenic phosphodiester mimic that supports RNase H recruitment. Phosphoryl guanidine (PN) replaces a non-bridging oxygen with a guanidino group, producing a largely charge-neutral, stereogenic linkage. Phosphonoacetate replaces a non-bridging oxygen with a carboxymethyl group, giving an anionic, stereogenic linkage. Bottom row: backbone-replacement scaffolds, in which the entire sugar–phosphate backbone is replaced by a neutral architecture. In phosphorodiamidate morpholino oligomers (PMOs), morpholine rings are joined by neutral phosphorodiamidate linkages; in peptide nucleic acids (PNAs), the backbone is a neutral N-(2-aminoethyl)glycine pseudopeptide bearing nucleobases through methylenecarbonyl linkers. Both scaffolds are nuclease-resistant and do not support RNase H cleavage. Throughout, “Base” denotes a generic nucleobase, bold wedges indicate the nucleobase on the β-face, wavy bonds denote continuation of the oligomer chain to the adjacent 3′/5′ residues; note that the PNA aeg backbone is achiral. Created with Chemdraw.

Phosphate-linkage and backbone-replacement chemistries represent major strategies to improve biological stability, pharmacokinetic behavior, and functional persistence of oligonucleotide therapeutics. Native phosphodiester linkages are susceptible to nuclease-mediated cleavage, motivating the development of chemistries that reduce enzymatic degradation while also tuning protein binding, hybridization behavior, tissue distribution, and compatibility with mechanism-specific requirements such as RNase H recruitment or steric blocking. These strategies span multiple chemical levels, from substitution at the phosphodiester linkage to replacement of the native sugar–phosphate scaffold. Phosphorothioate (PS) linkages retain the native sugar–phosphate framework while replacing a nonbridging oxygen with sulfur, whereas phosphorodiamidate morpholino oligomers (PMOs), peptide nucleic acids (PNAs), and related scaffold-replacement chemistries use distinct architectures that alter charge, geometry, and biological interactions. Accordingly, linkage-level modifications and backbone-replacement strategies should be evaluated not only by their ability to improve nuclease resistance but also by their effects on mechanism compatibility, cellular distribution, protein interactions, hybridization geometry, and therapeutic tolerability. These phosphate-linkage modifications, which retain the native sugar–phosphate framework, and the PMO and PNA scaffold-replacement classes, which do not, are summarized in Table 2 together with their charge characteristics, RNase H compatibility, and principal advantages and limitations (Figure 4).

### 3.1. Phosphorothioate Linkages and Stereochemical Control

Phosphorothioate (PS) linkages are one of the most established examples of linkage-level modification because they directly address an early limitation of therapeutic oligonucleotides: rapid nuclease-mediated degradation of the native phosphodiester backbone. In phosphorothioate-modified oligonucleotides, substitution of a nonbridging phosphate oxygen with sulfur increases resistance to nuclease-mediated degradation while retaining the native anionic backbone attribute. Its continued use reflects a practical design compromise: PS linkages can stabilize oligonucleotides without fully disrupting mechanism-specific activity, supporting RNase H recruitment in DNA gap regions of gapmer ASOs [96,97,98,99], and providing terminal exonuclease protection in chemically modified siRNA duplexes, although the magnitude of this effect depends on strand end, stereochemistry, and surrounding nucleotide chemistry [100,101]. However, PS substitution affects more than nuclease stability, the sulfur-containing linkage alters stereochemistry and protein-binding behavior, improving plasma protein association and tissue exposure in some contexts while also influencing potency, toxicity, and intracellular interactions depending on sequence, placement, and therapeutic modality [102,103,104,105]. This protein association is not incidental to the sulfur substitution; it is a large part of why PS oligonucleotides reach cells at all. Binding to cell-surface proteins routes the strand into receptor-mediated endocytic uptake, so that unformulated oligonucleotides lacking PS linkages are internalized poorly even when they carry 2′ modifications that confer affinity and stability [106]. PS therefore functions as a delivery-enabling chemistry as well as a stabilizing one, which is why it persists in nearly every systemically administered approved product despite lowering duplex stability against RNA.

The current role of PS chemistry is therefore less about applying backbone stabilization uniformly and more about understanding how PS behaves inside multi-modified therapeutic architectures. PS effects can change depending on whether the surrounding architecture is a gapmer ASO, splice-switching oligonucleotide, ligand-conjugated siRNA, or another chemically stabilized scaffold [107,108,109]. In ligand-conjugated siRNAs, Biscans et al. showed that extrahepatic efficacy depended on the combined scaffold design, including siRNA structure, PS content, linker composition, and hydrophobic conjugation, rather than PS content alone [110]. Similarly, Croft et al. showed that PS/2′-O-methyl single-stranded oligonucleotides can produce cytotoxicity in a sequence- and structure-dependent manner, emphasizing that sugar modification plus PS does not automatically resolve nonspecific interactions [111]. Studies using stereodefined or positionally varied PS designs show that the same sulfur substitution can produce different biological outcomes depending on where the PS linkage is placed and whether the phosphorus center adopts an Rp or Sp configuration [112,113]. In multi-modified oligonucleotides, where PS linkages are often combined with sugar modifications, ligand conjugates, or other backbone elements, the surrounding modifications may influence binding, delivery, or mechanism, but PS remains a large factor of how the strand engages nucleases, plasma and intracellular proteins, and mechanism-specific enzymes [114]. Together, these studies shift PS chemistry from a standalone stabilizing feature to a context-dependent design variable whose value depends on how backbone chemistry, sugar modification, conjugation, structure, and modality interact.

### 3.2. Alternative Phosphate-Linkage Chemistries

Alternative phosphate-linkage chemistries have emerged as PS solves one major problem while creating others. Although PS protects oligonucleotides from nuclease degradation, repeated sulfur substitution increases stereochemical heterogeneity and changes the protein-interaction surface of the strand, which can complicate potency, biodistribution, toxicity, and analytical control. Newer linkage analogues, including mesyl phosphoramidate, phosphoryl guanidine/phosphoryl guanidino, phosphorodithioate, boranophosphate, alkylphosphonate, phosphonoacetate, and related charge-modulating chemistries, attempt to alter the phosphate backbone more selectively by changing charge, polarity, hydrophobicity, or stereochemical behavior. Their value lies in whether they can separate desirable effects such as nuclease resistance and favorable pharmacokinetics from less desirable effects such as nonspecific protein binding, chemical heterogeneity, or toxicity. Thus, this class of modifications should be evaluated not as a search for a universal PS replacement, but as a set of backbone-engineering strategies for improving control over oligonucleotide behavior.

Mesyl phosphoramidate (MsPA) linkages illustrate how alternative phosphate-linkage chemistry can refine, rather than simply replace, PS-based design. Compared with PS, MsPA has been explored to reduce some liabilities associated with extensive sulfur substitution, including protein-binding-associated toxicity and pro-inflammatory effects, while preserving features important for RNase H1-active ASOs [115]. However, the strongest evidence supports MsPA as a positional modifier within PS gapmer architectures, not as a universal backbone replacement. Anderson et al. showed that replacing all PS linkages in a gapmer ASO with MsPA maintained chemical stability and RNA-binding affinity but reduced activity, whereas selected MsPA placement within PS gapmers improved potency, therapeutic index, inflammatory profile, and duration of effect [116]. Patutina et al. highlight a different advantage of MsPA chemistry: rather than simply substituting for PS as a nuclease-resistant linkage, MsPA-backbone ASOs targeting miR-21 showed improved RNA affinity and nuclease resistance while retaining RNase H-recruiting activity, suggesting that MsPA can strengthen target engagement when incorporated into an architecture that still supports productive cleavage [117]. By contrast, MsPA’s role in steric-blocking or splice-switching oligonucleotides appears less clearly superior to PS. Hammond et al. evaluated mesyl phosphoramidate oligonucleotides as potential splice-switching agents and found that a 2′-MOE mesyl oligonucleotide performed similarly to the corresponding PS oligonucleotide in SMA patient-derived fibroblasts but was inferior to nusinersen in a neonatal mouse survival study at the highest dose tested [118]. Thus, MsPA is best framed as a site-specific linkage tool that can reshape protein interaction, toxicity, and RNase H1-related performance in selected ASO designs, while its benefits in non-RNase H, steric-blocking modalities remain more architecture- and model-dependent.

Phosphoryl guanidine-containing, or PN, linkages represent a more targeted attempt to redesign the phosphate backbone by changing charge presentation rather than simply increasing sulfur substitution. Compared with PS, which retains an anionic backbone while introducing sulfur-dependent chirality and protein-interaction effects, phosphoryl guanidine-containing PN linkages replace a nonbridging oxygen with a nitrogen-containing, largely charge-neutral internucleotide group. This changes the backbone’s charge presentation and local interaction surface, allowing PN-containing oligonucleotides to alter nuclease resistance, duplex behavior, tissue exposure, and biological durability in ways that do not simply mirror PS substitution [119,120,121,122,123]. The therapeutic evidence base for PN/phosphoryl guanidine chemistry remains narrower than for PS but includes RNase H-active ASOs, stereopure splice-switching oligonucleotides, chimeric siRNA backbones, and phosphoryl guanidine gapmers, where PN placement has been evaluated against outcomes such as target knockdown, exon skipping, pharmacodynamic durability, tolerability, and disease-relevant functional phenotypes [124]. The narrower development of PN chemistry across oligonucleotide modalities may reflect more than limited exploration. The strongest available examples are concentrated in systems where backbone changes can be evaluated through direct functional readouts such as target reduction, splice correction, or RNAi-mediated silencing. Translation into modalities whose activity depends on higher-order folding, receptor recognition, catalytic geometry, or immune activation remains less established, including aptamers, CpG ODNs, DNAzymes/ribozymes, and other structurally constrained systems. In these contexts, PN-mediated changes in charge presentation, hydration, or local backbone geometry could improve nuclease resistance while also perturbing the structural or recognition features required for function. This makes the limited breadth of PN chemistry an important knowledge gap rather than simply a missing application area. Thus, PN chemistry is important not because it broadly replaces PS but because it illustrates how backbone charge and linkage identity can be engineered as modality-specific variables, while also highlighting that alternative phosphate-linkage chemistries remain unevenly validated across the wider oligonucleotide therapeutic landscape.

Other phosphate-linkage analogues, including alkylphosphonate and boranophosphate modifications, further illustrate how changing the phosphate backbone can alter charge, stability, and biological interactions, although the depth of therapeutic validation differs substantially across these chemistries. Migawa et al. demonstrated this clearly in gapmer ASOs, where site-specific replacement of selected PS linkages with charge-neutral alkylphosphonate linkages altered RNase H1 cleavage patterns while largely preserving activity, and replacement near the 5′ side of the DNA gap reduced or eliminated toxicity in several hepatotoxic ASOs [125]. In siRNA, Nikan et al. extended the same principle to a different architecture by showing that a single alkylphosphonate linkage in the guide-strand seed region improved specificity and therapeutic profile, with the strongest effects when placed at internucleotide positions 6–7 from the guide-strand 5′ end [126]. These studies show that neutral linkage chemistry is not simply useful because it reduces charge; its value comes from placing the neutral linkage where it changes an unwanted interaction without collapsing the productive mechanism. Boranophosphate studies make a related point from the opposite direction: Hall et al. showed that boranophosphate siRNAs retained RNAi activity while improving nuclease resistance and later reported that boranophosphate single-stranded siRNAs produced durable silencing activity that native ss-siRNA lacked [127]. Collectively, these studies show that alternative phosphate-linkage chemistries are best understood by the problem they are positioned to solve: alkylphosphonate substitutions can reduce charge-dependent interactions linked to toxicity or seed-mediated off-targeting, whereas boranophosphate linkages can improve nuclease resistance while preserving RNAi activity in selected scaffolds.

### 3.3. Backbone-Replacement Architectures: PMOs and PNAs

Backbone-replacement architectures represent a more substantial structural departure from native DNA or RNA than phosphate-linkage modification. Rather than altering the phosphodiester linkage while retaining the sugar–phosphate framework, PMOs and PNAs replace the native backbone with distinct synthetic scaffolds. PMOs use morpholine rings connected through neutral phosphorodiamidate linkages, whereas PNAs replace the sugar–phosphate backbone with a neutral pseudopeptide scaffold, commonly based on N-(2-aminoethyl)glycine units [128,129]. This distinction is important because these chemistries are not simply more nuclease-resistant versions of conventional oligonucleotides. Their neutral architectures change charge, geometry, and enzyme recognition enough to shift the kinds of therapeutic problems they are best positioned to address. PMOs are especially well aligned with steric-blocking mechanisms because their morpholino-phosphorodiamidate scaffold supports sequence-specific RNA binding without forming DNA/RNA-like heteroduplexes that efficiently recruit RNase H [130]. This makes PMOs useful for splice modulation, exon skipping, translational blocking, and related applications where the therapeutic goal is to redirect RNA processing or obstruct RNA interactions rather than degrade the transcript. Their clinical relevance is therefore strongest in contexts where durable steric blockade is sufficient to produce a functional outcome. At the same time, PMO performance is often limited by delivery and intracellular access rather than target-binding potential alone [131]. These delivery challenges should not be reduced to charge neutrality alone. Unconjugated PMOs may be stable in biological fluids, but steric-blocking activity requires productive intracellular and, for splice-switching targets, nuclear access rather than simple persistence at the tissue level [132]. Peptide-conjugated PMOs address this limitation by increasing cellular and tissue delivery, yet the peptide component also introduces new variables in tissue selectivity, endosomal escape, biodistribution, and dose-dependent tolerability [133]. Thus, PMO optimization is not primarily a question of further improving nuclease stability; it is a question of delivering enough intact oligomer to the intracellular compartment where RNA processing or translation can be physically redirected.

PNAs occupy a related but chemically distinct position within backbone-replacement architectures. Like PMOs, PNAs replace the native sugar–phosphate framework with a neutral synthetic scaffold; because PNA–RNA hybrids are not substrates for RNase H, their antisense activity is generally better aligned with occupancy-based or steric-blocking mechanisms than with endogenous nuclease recruitment [134,135]. However, PNAs are not simply PMO analogues with a different scaffold. Their pseudopeptide backbone supports high-affinity, sequence-selective recognition of complementary DNA or RNA and, in selected DNA contexts, strand-invasion behavior that distinguishes PNAs from PMO-like splice-blocking scaffolds. This gives PNAs a broader identity as recognition molecules for DNA- or RNA-targeting applications, rather than only steric blockers of RNA processing [136,137,138]. This has made PNAs useful in anti-miRNA, antigene, steric-blocking, and structured nucleic acid-targeting strategies, where the key advantage is stable sequence recognition rather than catalytic transcript degradation. At the same time, PNA’s advantages in binding strength and enzymatic stability do not automatically translate into intracellular activity. Sequence-dependent solubility, aggregation, poor cellular entry, and endosomal sequestration can limit how much active PNA reaches the intended DNA or RNA target, particularly in antisense, anti-miRNA, or antigene applications. Studies using peptide-, lipid-, or nanoparticle-assisted PNA delivery show that biological effects often require strategies that improve cellular entry, endosomal escape, or compartmental access, making delivery an enabling part of therapeutic PNA design rather than a secondary formulation detail [139,140,141,142]. Compared with PMOs, which have clearer clinical traction in exon-skipping applications, PNAs remain more unevenly developed as therapeutic scaffolds, with much of their value lying in applications where high-affinity recognition or strand invasion can justify the added delivery burden.

Together, PMOs and PNAs show why backbone replacement should be evaluated separately from linkage-level modification. Unlike PS, MsPA, PN, or other phosphate-linkage analogues, these scaffolds do not tune a nucleic-acid-like backbone; they replace it. This replacement can improve metabolic stability and support durable steric recognition, but it also changes the structural and charge features that govern enzyme recruitment, biomolecular association, uptake pathways, and intracellular distribution. As a result, PMOs are best established in steric-blocking modalities such as splice-switching and exon-skipping therapeutics, where sustained binding to pre-mRNA can redirect RNA processing without requiring transcript degradation. PNAs, by contrast, are more often positioned as high-affinity recognition scaffolds for antisense, anti-miRNA, antigene, and structured nucleic acid-targeting strategies, although their therapeutic translation remains more dependent on delivery-enabling modifications. The trade-off is therefore different from phosphate-linkage chemistry: PMOs and PNAs can be powerful when stable target occupancy is sufficient to produce the desired biological effect, but they are less naturally suited to mechanisms that require endogenous nuclease recruitment or catalytic turnover. Their therapeutic value is best judged by whether scaffold stability, binding behavior, and delivery requirements converge on the same biological outcome, rather than by stability alone.

### 3.4. Emerging Scaffold, Conjugate, and Hybrid Architectures

Emerging oligonucleotide architectures increasingly treat chemical modification as a distribution of functions across the full construct rather than as a single change to a base, sugar, or backbone. In these designs, one component may provide sequence recognition; another improves metabolic stability; another enables tissue or cellular delivery; and another controls activation, localization, or release. This architecture-level logic appears across several hybrid formats, including aptamer–siRNA chimeras, aptamer–drug conjugates (ApDCs), aptamer–oligonucleotide conjugates (ApOCs), peptide-conjugated PMOs (PPMOs), heteroduplex oligonucleotides (HDOs), cyclic antisense oligonucleotides (cASOs), circular morpholinos, and nucleic-acid nanostructures. Although constrained sugar chemistries such as tricyclo-DNA, LNA, and cEt are addressed more directly in Section 4, they are relevant here when incorporated into hybrid architectures that distribute recognition, stability, delivery, and activity across multiple chemical features rather than relying on one modification class alone. In these systems, chemical modification becomes a problem of architectural compatibility: recognition, stability, delivery, and activation can be distributed across different molecular components, but the benefit of modular design is lost if delivery, recognition, activation, or effector function fails under the biological conditions where the construct must operate.

This broader hybrid approach is especially valuable because different modalities fail for different reasons. For a steric-blocking oligonucleotide, strong nuclease resistance and sustained binding may be insufficient if the molecule does not reach the correct transcript compartment. For an aptamer conjugate, target binding must survive chemical attachment and cargo loading. For a DNAzyme, added stability is useful only if substrate recognition, metal-dependent catalysis, and product release remain intact. For nucleic-acid nanostructures, multivalency and programmability can improve avidity, delivery, or controlled release, but they also increase structural complexity, heterogeneity, and analytical burden. Hybrid architectures therefore expand the design space, but they also distribute failure points across the construct: each added module must preserve the function it was meant to support rather than simply increasing chemical complexity.

Several recent directions illustrate this shift from single-modification optimization to modular chemical architecture. Heteroduplex oligonucleotide (HDO) strategies show that even established steric-blocking scaffolds can be re-engineered through strand pairing: Hasegawa et al. used a lipid–ligand-conjugated complementary strand hybridized to a PMO to enhance splice-switching activity in a Duchenne muscular dystrophy (DMD) mouse model, with improved delivery to key tissues and restoration of internal deleted dystrophin expression [143]. Aptamer-based systems provide a second architecture-level strategy, where the aptamer acts as a cell-selective recognition domain rather than the sole therapeutic effector; Vasconcelos et al. designed a transferrin receptor (TfR)-targeted aptamer–siRNA conjugate against CEBPB and reported target knockdown and reduced tumor burden in metastatic pancreatic ductal adenocarcinoma (PDAC) and hepatocellular carcinoma (HCC) models [144]. More complex functional nucleic-acid hybrids extend this principle further: Yan et al. developed an aptamer–deoxyribozyme metal–nucleic acid framework for HER2-positive gastric cancer, combining aptamer-based targeting, DNAzyme functionality, and payload delivery within the same construct [145]. Cyclic and stimulus-responsive oligonucleotides add another layer by using topology as an activity gate; Wang et al. developed cathepsin B-activatable cyclic antisense oligonucleotides that suppressed target interaction until enzymatic uncaging and produced cell-selective gene knockdown in vitro and antitumor activity in a PC-3 tumor mouse model [146]. These architectures make clear that modularity is not inherently beneficial; each added chemical feature must improve one limiting step without creating a new bottleneck in delivery, recognition, catalysis, or activation.

The main challenge for emerging hybrid architectures is that modularity can obscure, rather than simplify, structure–activity relationships. A targeting ligand, complementary strand, cyclic constraint, catalytic domain, or supramolecular carrier may improve persistence, cell selectivity, intracellular access, or conditional activation, but each addition also creates another point where folding, protein association, release kinetics, biodistribution, or safety can change. Hybrid designs should therefore not be interpreted as a more advanced replacement for single-modification strategies. Hybrid architectures are most informative when the contribution of each module can be resolved experimentally. A targeting ligand should improve cell or tissue selectivity, a complementary strand should improve delivery or intracellular access without suppressing activity, a cyclic constraint should control activation without preventing timely release, and a catalytic or recognition domain should retain the function that justified its inclusion. The field’s challenge is therefore not simply building more elaborate constructs, but distinguishing architectures that create separable functional gains from those that introduce complexity without clarifying which component drives the biological outcome.

### 3.5. Matching Linkage Chemistry to Therapeutic Function

The examples above show that linkage and scaffold chemistries cannot be evaluated by a single universal metric such as nuclease resistance, binding affinity, or stability. The same chemical feature can be beneficial, neutral, or limiting depending on which molecular event must occur after target recognition. For RNase H-dependent ASOs, backbone chemistry must protect the oligonucleotide while preserving a duplex geometry and interaction surface compatible with enzyme-mediated RNA cleavage. For RNAi therapeutics, chemical stabilization is useful only if guide-strand loading, passenger-strand release, and Argonaute-mediated silencing remain productive. For steric-blocking and splice-modulating oligonucleotides, the priority shifts toward persistent target occupancy and access to the relevant transcript compartment, without requiring catalytic RNA degradation. For aptamers, ribozymes, and DNAzymes, the tolerance for modification is further constrained by folding, ligand or substrate recognition, metal coordination, and catalytic turnover. Thus, the function of a modification is not defined only by what it changes chemically, but by whether that change supports the next required step in the therapeutic mechanism.

This functional view also explains why modern oligonucleotide designs increasingly combine linkage, scaffold, sugar, ligand, and architecture-level modifications rather than relying on isolated substitutions. The advantage of combination design is not that each modification contributes an independent benefit but that different chemical features can be positioned to address different steps in the therapeutic sequence. PS linkages may improve stability and pharmacokinetic exposure, while constrained sugars, alternative linkages, neutral scaffolds, or delivery ligands may alter binding, toxicity, tissue access, or intracellular localization. However, these effects are not automatically additive. A modification that improves nuclease resistance can reduce enzyme compatibility; a conjugate that improves uptake can alter biodistribution or intracellular trafficking; and a scaffold that strengthens target occupancy can create delivery or solubility constraints. The practical challenge is therefore architectural compatibility: each modification must improve the intended limiting property without shifting the major bottleneck to another step in recognition, delivery, activation, or effector function.

Taken together, the backbone and scaffold modifications discussed in this section define the physical framework in which an oligonucleotide therapeutic operates. PS and related phosphate-linkage chemistries preserve a nucleic-acid-like scaffold while tuning nuclease resistance, charge distribution, protein association, and enzyme compatibility. Alternative linkages such as MsPA, PN-type, alkylphosphonate, and boranophosphate analogues show that changing the internucleotide linkage can redistribute these properties, but often only within narrow positional or architectural windows. PMOs and PNAs represent full scaffold-replacement strategies: replacing the native sugar–phosphate framework can produce strong metabolic stability and steric recognition, but it also shifts the design burden toward delivery, solubility, intracellular access, and compatibility with non-cleavage-based mechanisms. Hybrid architectures add another layer of design by combining backbone or scaffold chemistry with separate targeting, delivery, activation, or effector modules within the same therapeutic construct. The backbone is not merely a chemical support for nucleobase recognition; it establishes the interaction landscape through which the therapeutic strand can recruit enzymes, occupy RNA targets, fold catalytically, associate with proteins, or access intracellular compartments.

## 4. Sugar and Conformational Modification Strategies

**Figure 5:**
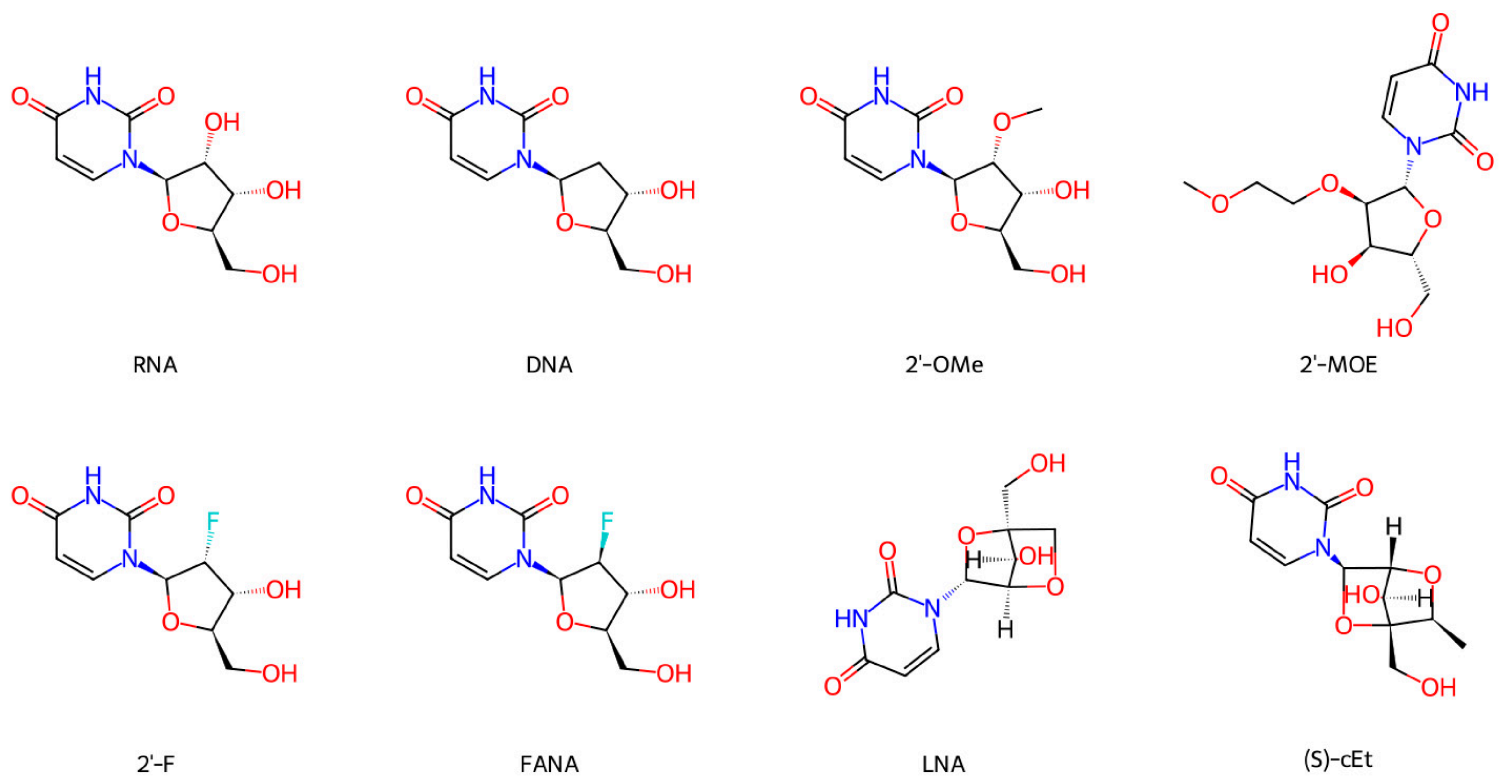
Sugar and conformational modifications for therapeutic oligonucleotides, grouped by structural class and drawn with explicit C2′ configuration. Top row—flexible 2′-substituted ribose analogues: the native 2′-hydroxyl is replaced by a 2′-O-methyl (2′-OMe), 2′-O-methoxyethyl (2′-MOE), or 2′-fluoro (2′-F) group. In all three, the substituent occupies the α-face (shown hashed), trans to the nucleobase, consistent with the ribo configuration; these modifications increase target affinity and metabolic stability and bias the sugar toward a C3′-endo (A-form) pucker, but do not support RNase H cleavage. The arabino-configured analogue 2′-fluoro-arabino nucleic acid (FANA) is shown alongside 2′-F to highlight that the two differ only by inversion at C2′: in FANA, the 2′-fluorine occupies the β-face (shown as a bold wedge), cis to the nucleobase. This stereochemical difference allows FANA, unlike the other sugars depicted, to behave as a DNA mimic whose hybrids with RNA support RNase H1-mediated cleavage. Bottom row—conformationally constrained sugars: locked nucleic acid (LNA) contains a 2′-O,4′-C methylene bridge that locks a rigid C3′-endo conformation and confers large gains in duplex affinity; constrained ethyl (cEt) carries the analogous bridge bearing an (S)-methyl group, providing comparable affinity with an improved tolerability profile; tricyclo-DNA (tcDNA) is a 2′-deoxy analogue rigidified by a 3′,5′-ethano bridge and a fused cyclopropane ring. As drawn (fully modified), these constrained sugars are used predominantly in steric-blocking and high-affinity gapmer-wing contexts and do not themselves support RNase H. Throughout, “Base” denotes a generic nucleobase, bold wedges indicate substituents on the β-face (toward the viewer) and hashed bonds the α-face, and wavy bonds denote continuation of the oligomer chain to the adjacent 3′/5′ residues. Created with Chemdraw 26.0.

Sugar and conformational modifications provide a second layer of control in oligonucleotide therapeutic design. Linkage and scaffold chemistries define the broader physical framework of the strand, including charge, backbone identity, protein association, and compatibility with enzyme- or steric-blocking mechanisms. Sugar modifications act more locally, tuning ribose geometry, duplex formation, nuclease susceptibility, target affinity, and conformational preorganization while retaining a nucleic-acid-like scaffold. Representative sugar modifications include flexible 2′-substituted ribose analogues such as 2′-O-methyl (2′-OMe), 2′-O-methoxyethyl (2′-MOE), and 2′-fluoro (2′-F); arabino-configured analogues such as 2′-fluoro-arabino nucleic acid (FANA) shown in Figure 5; and conformationally constrained analogues such as locked nucleic acid (LNA), constrained ethyl (cEt), and tricyclo-DNA (tcDNA). Their value is not simply that they make oligonucleotides more stable, but that they reshape the structural context in which binding, immune recognition, enzyme compatibility, and modality-specific activity occur. These sugar and conformational modifications are summarized in Table 3, grouped as flexible 2′-substituted ribose analogues, the arabino-configured analogue FANA, and conformationally constrained sugars, and noting that FANA—unlike the other modifications listed—supports RNase H-mediated cleavage.

### 4.1. 2′-Substituted Ribose Analogues: 2′-OMe, 2′-MOE, and 2′-F

2′-substituted ribose analogues modify the local chemistry of the ribose without replacing the nucleic-acid-like scaffold. In native RNA, the 2′-hydroxyl contributes to ribose hydration, conformation, and enzymatic recognition, but it also creates a chemical handle associated with ribonuclease susceptibility. Replacing this group with 2′-O-methyl (2′-OMe), 2′-O-methoxyethyl (2′-MOE), or 2′-fluoro (2′-F) changes both the steric and electronic environment around the sugar–phosphate backbone. These changes push the sugar toward a C3′-endo (A-form-like) conformation, which raises duplex stability and target affinity while reducing nuclease susceptibility; how far each of these shifts, and whether the net effect helps or hinders, is set by the bulk of the substituent and where it sits relative to the strand’s active region. The modality framework established in the Introduction is especially important for interpreting 2′-substituted sugars. In RNase H-dependent ASOs, 2′-OMe and 2′-MOE modifications are generally most useful in flanking regions, where they improve affinity and nuclease resistance while preserving a central DNA gap for RNase H-mediated cleavage [147,148,149]. In RNAi therapeutics, 2′-OMe and 2′-F modifications can be patterned more broadly across the duplex because the relevant constraint is not RNase H recruitment, but compatibility with Argonaute loading, strand selection, and target silencing [150,151]. In aptamers, DNAzymes, and other folded or catalytic oligonucleotides, the same modifications must be evaluated by whether they preserve the binding interface, substrate-recognition arms, metal-dependent catalytic geometry, or product-release behavior required for function [152,153,154]. The same local conformational control that makes 2′ substitution valuable in one modality can become a liability in another if it shifts the strand away from the geometry required for cleavage, loading, folding, or catalysis.

This placement sensitivity is especially apparent in multi-modified oligonucleotides, where 2′-substituted sugars are usually interpreted within a broader backbone, strand, or motif context rather than as isolated changes. In gapmer ASOs, 2′-modified wings are paired with PS or other linkage chemistries to increase binding and protect the strand while leaving the DNA gap available for RNase H-mediated cleavage [155,156]. In siRNAs, 2′-OMe and 2′-F modifications are commonly patterned across the guide and passenger strands to improve metabolic stability and reduce innate immune activation while preserving the strand selection, Argonaute loading, and target-silencing steps required for RNAi activity [157,158,159]. For folded or catalytic oligonucleotides, placement becomes more restrictive: sugar modification of substrate-recognition arms can improve stability or target access, whereas modification of catalytic cores, binding interfaces, or conformational switches can either weaken or enhance activity depending on whether the altered sugar geometry preserves the structural transitions required for target binding and cleavage [160,161,162,163]. The value of 2′-substituted sugars therefore lies in their positional specificity: the same local change can reinforce nuclease protection, binding, immune compatibility, or enzyme engagement at one site, while disrupting folding, catalytic geometry, or productive protein interactions at another.

The interaction between 2′ sugar chemistry and backbone chemistry is especially important because both modifications can affect the same biological interface. Shen et al. showed that 2′-F-modified PS oligonucleotides reduced levels of the DBHS proteins P54nrb and PSF, and later work with 2′-F gapmer PS-ASOs linked hepatotoxicity in mice to intracellular protein binding and DBHS protein loss [164]. In this case, the PS backbone provides the protein-interacting scaffold, while the 2′-F sugar pattern changes the intracellular protein-interaction profile and toxicity outcome. Choung et al. evaluated siRNAs containing 2′-OMe, 2′-F, and phosphorothioate modifications and showed that selected terminal modification patterns improved serum stability while retaining RNAi efficacy [165], Wu et al. showed that 2′-OMe-phosphorodithioate-modified siRNAs increased RISC loading and enhanced antitumor activity, illustrating how a combined sugar–backbone modification can affect RNAi performance beyond nuclease stabilization alone [166]. Even for established chemistries such as 2′-OMe, 2′-MOE, and 2′-F, therapeutic performance still depends on architectural context. When combined with backbone chemistries, the same local sugar change that improves metabolic stability or immune compatibility can also reshape protein binding, RISC loading, or effector-complex engagement.

### 4.2. Arabino-Configured Sugar Analogues: FANA and Related Systems

Arabino-configured sugar analogues expand the sugar-modification discussion beyond simple 2′ substitution by changing the stereochemical relationship between the sugar, nucleobase, and backbone. 2′-Fluoro-arabino nucleic acid (FANA) illustrates this distinction because it contains a 2′-fluoro substituent in the arabino sugar configuration, rather than the ribose configuration used in 2′-F RNA; this stereochemical difference changes the local sugar geometry and can alter duplex conformation and RNase H processing [167,168]. This seemingly local stereochemical change can alter duplex conformation, nuclease resistance, and compatibility with enzyme-mediated processes such as RNase H cleavage or RNAi activity, making FANA more than a fluorinated variant of 2′-substituted RNA [169,170]. Its value depends on whether the arabino geometry supports the structural requirement of the modality: in antisense contexts, Takahashi et al. showed that self-delivering FANA oligonucleotides can act through RNase H1 recruitment or steric blockade against HIV-1 RNA targets, while Fakih et al. showed that placing FANA antisense strands in spherical nucleic acid architectures improved stability, cellular uptake, and gene-silencing potency relative to free FANA [171,172]. In RNAi systems, FANA substitution has been shown to improve siRNA serum stability while retaining compatibility with intracellular RNAi machinery; in functional nucleic acid systems, FANA-based enzymes demonstrate that arabino-configured sugars can support substrate recognition and RNA-cleavage catalysis when incorporated into appropriately selected catalytic architectures [173,174]. FANA therefore shows that sugar-modification effects are governed not only by the substituent introduced but by the stereochemical geometry through which that substituent is presented to the duplex, enzyme, or catalytic architecture.

FANA’s modality range is best illustrated by studies that move beyond simple duplex stabilization. Rose et al. selected fully modified FANA aptamers that bound HIV-1 integrase with picomolar affinity, showing that arabino-configured sugars can support folded recognition architectures rather than only antisense hybridization [175]. Alves Ferreira-Bravo et al. extended this recognition logic to SARS-CoV-2 by selecting FANA aptamers against the spike receptor-binding domain that blocked ACE2 binding, demonstrating that FANA scaffolds can support protein-targeting function in folded XNA aptamers [176]. In catalytic systems, Wang et al. evaluated an RNA-cleaving FANAzyme and showed that a fully FANA enzyme can support substrate recognition and catalytic RNA cleavage [177], while Liu et al. further expanded the enzymatic toolkit available for FANA manipulation [178]. FANA therefore occupies a distinct position in the sugar-modification landscape: it is not as broadly established as 2′-OMe, 2′-MOE, 2′-F, or LNA-like chemistries, but it shows that changing sugar stereochemistry can produce functional behavior that cannot be inferred from the presence of a 2′-fluoro substituent alone. Its significance lies in demonstrating that sugar configuration can be used to tune whether an oligonucleotide behaves as an antisense strand, RNAi-compatible duplex, folded aptamer, or catalytic XNAzyme scaffold.

### 4.3. Conformationally Constrained Sugars: LNA, cEt, and tcDNA

Conformationally constrained sugar analogues occupy a different position from flexible 2′-substituted sugars because they do not merely change the substituent attached to the ribose; they restrict the sugar–phosphate framework into geometries that can preorganize the strand for target recognition. Locked nucleic acid (LNA), constrained ethyl (cEt), and tricyclo-DNA (tcDNA) all exploit this principle, but they do so with different translational profiles. LNA and cEt have been especially influential in high-affinity ASO gapmer design, where constrained sugar wings can improve potency by strengthening target binding, but high affinity can also increase the risk of sequence-dependent off-target effects or toxicity when not carefully controlled [179,180,181,182]. tcDNA, by contrast, has been explored largely as a steric-blocking or splice-modulating scaffold when used as a fully modified oligonucleotide because fully modified tcDNA does not support RNase H activity and is instead suited to exon skipping, exon inclusion, correction of aberrant splice sites, or prevention of protein binding. A notable body of work has applied this chemistry to Duchenne muscular dystrophy and related muscular dystrophy models, where systemic tcDNA ASOs have been evaluated for tissue exposure, exon-skipping activity, dystrophin restoration, and safety [183,184,185]. Thus, conformational constraint is best understood as an affinity- and geometry-amplifying strategy: it can lower the structural cost of productive target binding, but the same preorganization can become a liability when excess affinity, reduced flexibility, or altered protein engagement interferes with cleavage, turnover, delivery, or tolerability.

Several studies show that conformationally constrained sugars do not act as isolated affinity boosters once incorporated into therapeutic architectures; their effects are shaped by the backbone chemistry, target site, tissue context, and mechanism of action in which they are placed. For example, in PS gapmer ASOs, high-affinity constrained sugar wings can improve target engagement, but the safety profile is not determined by affinity alone. Studies of LNA-modified gapmers show that hepatotoxicity can be sequence-associated and, in some cases, linked to RNase H1-dependent, off-target reduction in partially complementary or very long transcripts. Stronger binding can amplify unintended cleavage events when the modified architecture stabilizes nonproductive interactions [186,187]. Rather than treating LNA or related constrained sugars as inherently toxic or inherently superior, recent work has emphasized that constrained-gapmer safety depends on sequence selection, hybridization-dependent off-target assessment, transcriptome-wide screening, and chemistry-level mitigation strategies such as stereochemical or nucleobase modification [188,189,190]. cEt-containing ASOs illustrate a related use of conformational preorganization within PS gapmer architectures: the constrained sugar can support high-affinity target binding and potent RNase H-dependent knockdown, but its performance depends on how cEt residues are positioned relative to the DNA gap and how the sequence balances potency, protein interactions, and tolerability [191,192]. Fully modified tcDNA shifts the design logic away from RNase H-mediated cleavage toward steric-blocking applications, including splice modulation, because fully modified tcDNA oligomers are not designed to recruit RNase H and instead act through durable RNA occupancy [193,194,195]. Together, these examples show that conformational constraint changes the energetic and structural terms of oligonucleotide recognition; whether that improves therapeutic performance depends on if stronger preorganization supports the intended output or stabilizes an interaction the modality cannot tolerate.

### 4.4. Sugar-Modification Combinations and Function-Dependent Design Across Therapeutic Modalities

Sugar-modification patterns are best interpreted through the therapeutic modality in which they are used. A sugar chemistry that increases affinity, nuclease resistance, or conformational preorganization can improve one modality while weakening another if it changes the structural interaction required for activity. In RNase H-dependent ASOs, sugar modifications are typically positioned to strengthen and protect flanking regions while preserving a central DNA-like gap that forms the enzyme-compatible heteroduplex required for RNase H-mediated cleavage. In RNAi therapeutics, broader 2′-OMe and 2′-F patterning can be tolerated because the critical requirement is compatibility with duplex loading, strand selection, and Argonaute-mediated silencing rather than RNase H recruitment. In steric-blocking oligonucleotides, aptamers, decoys, and catalytic nucleic acids, sugar modifications must instead be judged by whether they preserve target occupancy, protein-recognition geometry, folding, substrate binding, or catalytic turnover. The same sugar modification therefore cannot be assigned a fixed therapeutic meaning outside the architecture in which it is placed.

Combination designs are often favored because no single sugar modification optimizes all required properties simultaneously. A 2′-MOE, LNA, or cEt wing can increase affinity in a gapmer ASO, but this benefit depends on pairing the modified wing with a cleavage-compatible DNA gap and a backbone chemistry that supports pharmacokinetic exposure without excessive protein-mediated liability. The sugar modification is not the RNase H-recruiting feature; rather, it helps define the binding and stability context around the DNA gap that permits cleavage. In siRNA, a 2′-OMe or 2′-F pattern can improve metabolic stability and reduce immune stimulation, but the duplex must still present an Argonaute-compatible guide strand and appropriate passenger-strand behavior. FANA, LNA, cEt, and tcDNA further show that stronger preorganization or altered sugar geometry is valuable only when it reinforces the intended functional interaction. These chemistries can support durable target occupancy, RNAi compatibility, folded recognition, or splice modulation, but they can also create liabilities if they stabilize off-target binding, alter catalytic geometry, reduce turnover, or increase delivery and tolerability burdens. The field has therefore moved toward patterned sugar modification rather than uniform substitution: the key issue is not which sugar is intrinsically superior, but whether each modified position has a defined functional role.

Hybrid architectures make this interpretation even more important because sugar chemistry may no longer be responsible for only target binding or nuclease protection. In heteroduplex oligonucleotides, peptide- or ligand-conjugated steric blockers, spherical nucleic acids, aptamer-guided constructs, cyclic oligonucleotides, and stimulus-responsive systems, sugar modifications operate alongside delivery modules, complementary strands, topology, or carrier scaffolds. In these settings, improved affinity or nuclease resistance is useful only if the modified strand reaches the correct compartment and remains available for the intended interaction. A sugar modification that strengthens target binding may add little value if delivery remains limiting, while a delivery-enhanced architecture may fail if the sugar pattern disrupts folding, strand loading, catalytic release, or effector recruitment. Sugar chemistry is therefore an architectural variable rather than a standalone optimization step.

Across modalities, sugar modifications do not simply improve oligonucleotides; they redistribute the constraints that determine whether a therapeutic strand can act. In RNase H-dependent systems, sugar modifications must be positioned so they do not replace or distort the DNA-like gap required for enzyme-mediated cleavage. In occupancy-based systems, they must sustain target binding without creating delivery, solubility, or tolerability barriers. In folded or catalytic systems, they must stabilize the active architecture without freezing the structural transitions required for recognition, cleavage, or product release. Sugar-modification combinations are most valuable when they are mapped onto the functional sequence of the modality: delivery, target engagement, effector recruitment, activation, turnover, and release.

## 5. Design Trade-Offs and Therapeutic Selection Principles

### 5.1. Clinically Established Chemistry–Modality Pairings

Clinically successful oligonucleotide modifications are those that overcome pharmacological barriers and preserve the mechanism of action. As of March 2024, Vinjamuri et al. identified 20 U.S. Food and Drug Administration (FDA)- and European Medicines Agency (EMA)-approved commercial oligonucleotide drug products: 1 aptamer, 12 antisense oligonucleotides (ASOs), 6 small interfering RNAs (siRNAs), and 1 mixed single-/double-stranded polydeoxyribonucleotide product [196]. Since that review, additional FDA approvals have expanded the clinical landscape, including imetelstat and olezarsen in 2024 and fitusiran, donidalorsen, and plozasiran in 2025, bringing the field to roughly two dozen approved oligonucleotide products. The FDA’s 2024 clinical pharmacology guidance further reflects the maturation of oligonucleotide therapeutics as a regulated drug class, emphasizing systematic evaluation of immunogenicity, hepatic and renal impairment, corrected QT interval (QTc) prolongation risk, and drug–drug interaction potential [197]. Modifications seeing clinical success have typically encompassed a smaller set of chemistry–modality pairings: PS and 2′-sugar-modified ASOs, PMO steric blockers, and chemically stabilized siRNAs delivered by lipid nanoparticles or ligand conjugation. This pattern separates chemistries that are clinically established from those that remain promising but experimentally concentrated.

Table 4 maps the approved products onto their chemistry and delivery platform and makes this clustering explicit. Despite the breadth of modification chemistry surveyed in Section 3 and Section 4, the approved landscape is built almost entirely from phosphorothioate linkages, 2′-F, 2′-O-methyl, 2′-O-MOE, and the morpholino scaffold, and every systemically administered product depends on either lipid nanoparticle formulation or GalNAc conjugation to reach its target tissue. The table therefore functions less as a clinical catalogue than as a record of which chemistry–modality–delivery combinations have survived translation.

Among approved oligonucleotide modalities, ASO therapeutics provide one of the clearest examples of how backbone and sugar modifications are paired to meet clinical pharmacology demands. Approval of an oligonucleotide therapeutic requires more than showing that a modified sequence can modulate RNA in experimental systems. The chemical architecture must support the intended mechanism while also producing a molecule that can be dosed repeatedly, distributed predictably, manufactured reproducibly, and monitored for safety in patients. ASOs are a useful first example because they represent the largest approved-product category and show how the same broad modification families can produce different clinical mechanisms depending on architecture. Nusinersen (Spinraza) uses a fully modified 2′-O-methoxyethyl phosphorothioate (2′-MOE/PS) design to promote durable pre-mRNA occupancy and alter splicing without relying on RNase H-mediated cleavage. Inotersen (Tegsedi) also uses 2′-MOE/PS chemistry, but as an RNase H-dependent gapmer: modified wings increase affinity and stability, while the central DNA-like gap enables target RNA cleavage. This contrast translates the chemistry discussed earlier into a clinical design principle: PS and 2′-MOE do not define the mechanism by themselves; the placement pattern determines whether the therapeutic strand supports splice correction or transcript degradation.

Volanesorsen (Waylivra) provides a useful case study for the safety side of the same 2′-MOE/PS gapmer platform. Like inotersen, volanesorsen uses a PS-modified gapmer architecture to reduce target RNA through RNase H-mediated cleavage, but its clinical use has been shaped by thrombocytopenia risk and the need for platelet monitoring. This does not mean that the PS backbone alone explains the clinical safety profile. Rather, volanesorsen illustrates a broader translational point: once an ASO is chemically engineered for systemic persistence and potent target reduction, approval and clinical use depend on whether protein interactions, immune effects, tissue exposure, renal or hepatic burden, and hematologic monitoring can be managed in patients. In this sense, the same chemistry that makes systemic gapmer ASOs pharmacologically viable also creates a clinical-development burden that cannot be resolved by potency data alone.

PMO therapeutics provide a contrasting clinical example because they achieve nuclease resistance and steric blockade through scaffold replacement rather than through PS/2′-sugar modification. Eteplirsen (Exondys 51), golodirsen (Vyondys 53), viltolarsen (Viltepso), and casimersen (Amondys 45) are phosphorodiamidate morpholino oligonucleotides approved for exon-skipping applications in Duchenne muscular dystrophy, with each designed to redirect pre-mRNA splicing rather than degrade the transcript. Their neutral morpholino-phosphorodiamidate scaffold supports this RNase H-independent mechanism by allowing durable RNA occupancy while resisting nuclease degradation. However, the PMO approvals also show that replacing the backbone does not remove the translational barriers faced by oligonucleotide therapeutics. PMOs can be chemically stable and mechanistically appropriate for steric blockade, yet their clinical performance still depends on tissue exposure, dose, intracellular and nuclear access, renal safety monitoring, and whether exon skipping produces enough functional protein restoration to support clinical benefit. Thus, PMO drugs illustrate a different version of the same principle seen with ASOs: chemical architecture can make a mechanism possible, but approval and clinical use depend on whether the remaining delivery and safety barriers are manageable in the target tissue.

RNAi therapeutics provide an even clearer example of how clinical translation depends on combining chemical stabilization with delivery design. Patisiran (Onpattro), the first FDA-approved RNAi therapeutic, is a double-stranded siRNA formulated as a lipid complex for hepatocyte delivery; its structure includes 2′-O-methyl nucleotides, illustrating how sugar modification was incorporated into an RNAi architecture that still had to be packaged in a lipid nanoparticle-like formulation to reach the liver. Vutrisiran (Amvuttra) shows the next stage of this design logic: it is a transthyretin-directed siRNA administered subcutaneously once every three months, and its structure includes triantennary N-acetylgalactosamine (GalNAc) residues that enable hepatocyte delivery. Together, these products show that clinically successful siRNAs are not simply stabilized duplexes. Their sugar and backbone modification patterns must preserve Argonaute-mediated silencing, while the delivery architecture determines whether the duplex reaches the tissue where that silencing can occur. The movement from lipid-complex delivery with patisiran to GalNAc-conjugated subcutaneous siRNAs such as vutrisiran also illustrates how clinical RNAi development has shifted toward combining 2′ sugar stabilization, strand-biasing chemistry, and tissue-selective delivery to support durable dosing schedules and manageable systemic exposure.

The approved-product landscape therefore provides a practical filter for interpreting the modification strategies discussed in this review. PS/2′-sugar ASOs, PMOs, and chemically stabilized siRNAs have translated because their chemistries solve specific clinical-development problems: systemic exposure for ASOs, nuclease-resistant steric blockade for PMOs, and hepatocyte-directed delivery for siRNAs. However, this same landscape also shows that many chemically sophisticated designs remain experimentally concentrated rather than clinically established. FANA, tcDNA, MsPA, PN-type linkages, stereodefined PS designs, cyclic oligonucleotides, aptamer-guided conjugates, DNAzyme/XNAzyme systems, and nucleic-acid nanostructures all expand the modification space, but they have not yet produced the same breadth of approved therapeutic products. Their translational challenge is not merely to outperform established chemistries in binding, stability, or potency but to show that the added chemical complexity improves delivery, safety, manufacturability, or mechanism-specific activity in a way that can survive clinical evaluation. This distinction provides the basis for the next section: experimental chemistries should be judged not only by what they enable in model systems, but by what translational barrier they are positioned to solve.

### 5.2. Experimental Chemistries and the Translational Gap

The limited clinical representation of many emerging chemistries reflects the difference between demonstrating a useful molecular property and establishing a therapeutic platform. Many chemistries discussed in this review, including FANA, tcDNA, MsPA, PN-type linkages, stereodefined PS designs, cyclic oligonucleotides, aptamer-guided conjugates, DNAzymes, XNAzymes, and nucleic-acid nanostructures, have demonstrated useful properties in experimental settings but remain less represented among approved therapeutics than PS/2′-modified ASOs, PMOs, and stabilized siRNA platforms. The gap is therefore not simply chemical performance; it is whether the modified architecture can show reproducible delivery, predictable tissue exposure, acceptable safety margins, scalable manufacturing, and a mechanism-specific pharmacodynamic readout that regulators and clinicians can interpret.

DNAzyme therapeutics illustrate this distinction because they have reached human studies but have not become an approved therapeutic class. Dz13, a 10–23 deoxyribozyme targeting JUN/c-jun mRNA, was evaluated by single intratumoral injection in a phase 1 first-in-human trial for nodular basal-cell carcinoma and was reported to be safe and well tolerated at all tested doses, with no detectable systemic Dz13 exposure and reduced c-Jun expression in excised tumors. Public descriptions of this trial emphasize local administration and safety/tolerability rather than an extensively chemically modified clinical architecture, so Dz13 is best interpreted as evidence that a DNAzyme can be delivered locally and produce target-associated pharmacodynamic effects in humans, not as evidence that systemic DNAzyme delivery has been solved. SB010, an inhaled GATA3-specific DNAzyme, provides a second and more advanced local-delivery example: a phase 2a allergen-challenge study reported improved lung-function responses after inhaled treatment, supporting that a DNAzyme can produce measurable clinical activity when delivered directly to the relevant tissue. Together, these studies show that DNAzyme translation has achieved more than in vitro proof-of-concept; local or topical administration can partially bypass systemic delivery barriers and allow target engagement to be evaluated in humans. The unresolved translational question is broader platform generalization. For DNAzymes to move beyond selected local-delivery settings, future candidates must demonstrate that chemical stabilization, intracellular access, target accessibility in structured RNA, catalytic metal availability, product release, and immune or off-target effects remain compatible with repeated dosing in the intended tissue.

A similar distinction applies to emerging sugar, linkage, and scaffold chemistries. FANA, tcDNA, MsPA, PN-type linkages, stereodefined PS designs, and cyclic or stimulus-responsive oligonucleotides should not be grouped together as if they face the same translational barrier. Some remain primarily chemistry-enabling tools, others have advanced into disease-model studies, and others have begun to address clinically relevant safety or delivery questions. FANA and XNAzyme systems, for example, show that alternative sugar configurations can support hybridization, recognition, and catalysis, but they have not yet reached the same clinical position as 2′-MOE, 2′-OMe, 2′-F, or PMO chemistries that appear in approved products. tcDNA has produced strong experimental evidence in steric-blocking and splice-modulating designs, including systemic tissue-exposure advantages in preclinical neuromuscular models, but it remains better described as a developing scaffold than as a clinically established analogue of PMO. Alternative linkage strategies such as MsPA, PN-type linkages, and stereodefined PS designs are closer to optimization tools for known ASO or siRNA architectures: their translational value will depend on whether they reduce specific liabilities such as toxicity, protein-binding heterogeneity, or duration limits without increasing manufacturing and characterization burden. In this sense, emerging chemistries are not behind simply because they are newer; they are at different stages of proving whether their chemical advantages remain meaningful under clinical-development constraints.

The translational gap is especially pronounced for modalities whose activity depends on folded structure, target recognition, or catalytic turnover rather than relatively direct duplex-mediated mechanisms. ASOs and siRNAs benefit from pharmacodynamic readouts that are comparatively straightforward to connect to mechanism, such as target RNA reduction, splice correction, or protein lowering. By contrast, aptamers, decoys, DNAzymes, ribozymes, XNAzymes, and hybrid nanostructures often require additional evidence that the modified construct folds correctly, reaches the relevant compartment, engages the intended target, remains active under physiological conditions, and releases or turns over product when required. A DNAzyme or XNAzyme may show efficient cleavage in vitro, but clinical translation requires evidence that substrate recognition, catalytic metal availability, intracellular localization, and degradation kinetics remain favorable in vivo. Similarly, an aptamer conjugate may bind a target in vitro, but its clinical value depends on whether conjugation, cargo loading, circulation, and tissue exposure preserve the binding interface and therapeutic payload function.

Experimental chemistries should therefore be evaluated by the translational problem they are positioned to solve, not by novelty alone. A new linkage, sugar, scaffold, or hybrid architecture is most compelling when it addresses a defined barrier that has limited existing modalities, such as poor tissue exposure, excessive protein binding, short duration, weak target occupancy, immune activation, or uncontrolled activity. However, the same modification becomes less meaningful if it improves an isolated in vitro property while adding uncertainty in delivery, safety, analytical characterization, or manufacturability. This distinction is important for interpreting the next stage of oligonucleotide engineering: the field does not need more chemical diversity in the abstract; it needs clearer evidence connecting chemical placement to clinically relevant outcomes across modalities. That need provides the basis for modality-specific selection principles, where a modification is judged by whether it improves the limiting step of a therapeutic architecture without compromising the molecular event responsible for activity.

### 5.3. Selection Principles Across Therapeutic Modalities

The transition from experimental chemistry to clinical candidate requires evidence that the modification improves a translational barrier, not just a molecular property. A new sugar, linkage, scaffold, or hybrid architecture should therefore be evaluated against the same categories that have shaped approved oligonucleotide drugs: mechanism-linked pharmacodynamics, tissue exposure, safety monitoring, manufacturability, and analytical control. Safety evidence for emerging modifications has to be tied to the specific interaction that the chemistry changes. PS linkages help systemic ASOs persist by increasing nuclease resistance and biomolecular association, but that same sulfur-containing backbone also changes plasma and intracellular protein interactions during repeated dosing. High-affinity gapmer designs containing LNA, cEt, or related constrained sugars raise a different safety question: stronger hybridization can improve target knockdown, but it can also stabilize partial off-target pairing enough to support unintended RNase H1-mediated cleavage. 2′-F/PS combinations add another example because the sugar–backbone pairing can alter intracellular protein interactions rather than simply improving stability. For an emerging linkage such as MsPA, PN-type chemistry, stereodefined PS, or a cyclic architecture, the translational question is therefore chemistry-specific: which liability does the new design reduce, and does that improvement persist when the molecule is evaluated in vivo?

Delivery evidence should be interpreted with the same chemistry-specific standard. PMO approvals show that a neutral, nuclease-resistant scaffold can support exon-skipping therapy, but they also show that scaffold stability does not automatically solve tissue access, intracellular uptake, or nuclear delivery. RNAi therapeutics make the opposite point: chemically stabilized siRNA duplexes became broadly clinically useful only when paired with delivery systems that made hepatocyte exposure predictable, first through lipid-complex delivery and then through ligand-directed approaches such as GalNAc conjugation. For emerging scaffolds such as PNA, tcDNA, FANA, DNAzymes, XNAzymes, and cyclic oligonucleotides, the translational question is therefore not whether the chemistry improves stability or binding in isolation, but whether it produces a molecule that reaches the compartment where its mechanism can operate. This is where experimental potency often diverges from clinical readiness: activity in a controlled model system may not translate if the modified construct cannot achieve sufficient exposure in the relevant tissue without an additional delivery architecture.

Selection also depends on which property is being optimized, and the comparative data answer that question more precisely than a general preference for stability would suggest. Modification at the 2′-position raises pairing stability with complementary RNA by approximately 1 °C per residue for both 2′-O-methyl and 2′-O-MOE, while substituents such as 2-fluoroethyl, 2-(imidazol-1-yl)ethyl, and 2-[(N,N-dimethylamino)oxy]ethyl reach roughly 1.5 °C per residue, and conformational locking in LNA raises Tm by as much as 10 °C per modified nucleotide. Nuclease resistance follows a different order, and one that is not a simple function of steric bulk: the bulky 2-(benzyloxy)ethyl substituent affords very little protection against 3′-exonuclease attack, whereas zwitterionic substituents such as 2-(imidazol-1-yl)ethyl and 2-[(N,N-dimethylamino)oxy]ethyl rank among the most protective, an effect attributed to positioning a positive charge near the 3′-phosphate rather than to occlusion of the linkage. Phosphorothioate substitution, for its part, lowers Tm against RNA by about 1 °C per linkage, and its diastereomers divide the two properties between them, since the Sp isomer resists 3′-exonuclease degradation better than Rp yet forms the less stable duplex with RNA. That these orderings fail to coincide is more instructive than any individual ranking, because neither one predicts which chemistries became drugs. The substituents that lead on affinity and on nuclease resistance are not those that reached patients: approved oligonucleotide therapeutics rest on a small set of early chemistries, namely 2′-F, 2′-O-methyl, phosphorothioate, 2′-O-MOE, and the neutral PMO backbone. LNA makes the divergence explicit, since LNA-containing ASOs are five- to tenfold more potent than MOE equivalents in animal studies, yet renal and hepatic toxicity has constrained their clinical progress. The operative question for a modification is therefore not which property it maximizes, but whether the property it maximizes is the one the intended mechanism requires [198].

Analytical control becomes more important as modification strategies become more architectural. A uniformly modified ASO or PMO already requires rigorous identity, purity, and impurity profiling, but hybrid designs add additional layers that must be defined before clinical translation is credible. Stereodefined PS designs must control phosphorus configuration; ligand-conjugated siRNAs and ASOs must define conjugation identity and linker stability; cyclic or stimulus-responsive oligonucleotides must distinguish the inactive, activated, and degradation states; and aptamer-guided or nanostructured systems must show that the assembled construct is reproducible rather than a mixture of partially functional species. The translational issue is not that these architectures are too complex to develop, but that their added function has to remain measurable and manufacturable at the same time.

The clinical-development standard for emerging modifications is not whether they appear more advanced than established chemistries, but whether their added function remains visible under translational pressure. A new sugar, linkage, scaffold, or hybrid architecture should be able to show what problem it solves in vivo, what mechanism it preserves, and what new uncertainty it introduces. This framing helps explain why approved oligonucleotide products cluster around a smaller number of modification families despite the breadth of experimental chemistry available. The next challenge is to make modification selection less dependent on late-stage empirical filtering and more grounded in predictive relationships between chemistry, mechanism, and in vivo performance.

## 6. Future Directions in Chemically Engineered Oligonucleotide Therapeutics

The future of chemically engineered oligonucleotide therapeutics will not be defined by adding more modifications to already complex molecules but by learning which chemical combinations produce interpretable and clinically durable function. Approved ASO, PMO, and siRNA products show that successful translation usually depends on coordinated modification patterns rather than single chemical substitutions. However, the next challenge is no longer recognizing that combinations matter; it is determining when a combination creates a predictable therapeutic architecture and when it obscures the relationship between chemistry, mechanism, safety, and clinical performance. This distinction is especially important as emerging designs incorporate stereodefined linkages, alternative sugar geometries, scaffold replacement, ligand conjugation, cyclic activation, aptamer-guided delivery, or catalytic nucleic acid components. Future work must therefore move beyond asking whether a modification improves stability, affinity, or potency and toward asking whether the modified architecture remains mechanistically interpretable as it moves from controlled experimental systems into clinical development.

### 6.1. From Modification Catalogs to Predictive Design Rules

A major future direction is the development of predictive design rules that connect chemical modification patterns to modality-specific outcomes. Current modification knowledge remains fragmented across chemistry type, target sequence, delivery route, and therapeutic platform. As a result, design decisions are still often made through empirical screening rather than through models that can predict how a given modification pattern will affect RNase H compatibility, Argonaute loading, folded recognition, catalytic turnover, protein binding, immune activation, or tissue exposure. This limitation is especially important for multi-modified oligonucleotides, where the effect of one modification may depend on the placement and chemistry of neighboring modifications rather than on its isolated chemical identity.

Predictive design must also be modality-specific because each therapeutic class fails for different reasons. For RNase H-dependent ASOs, useful models need to connect sequence, chemical patterning, target-site accessibility, RNase H activity, off-target transcript reduction, protein binding, and in vivo tolerability. Early tools such as ASOptimizer suggest how deep learning may help design chemically modified RNase H-dependent ASOs and optimize ASO chemical diversity, but these approaches will be most valuable when they predict not only potency but also liabilities created by a given modification pattern. For splice-switching ASOs, the prediction target changes: models such as eSkip-Finder point toward the need to predict exon-skipping outcomes rather than RNA degradation, meaning that chemistry must be evaluated by occupancy, splice-site context, and nuclear activity. For siRNAs, predictive models must account for guide/passenger strand asymmetry, 2′-sugar patterning, terminal stabilization, delivery chemistry, and Argonaute-compatible silencing; recent machine learning studies that incorporate sequence and chemical modification patterns represent an important step toward this goal. Folded and catalytic modalities remain less developed computationally. Aptamers, decoys, DNAzymes, ribozymes, and XNAzymes require models that can evaluate folded recognition, substrate accessibility, metal-compatible catalytic geometry, turnover, and product release rather than duplex stability alone.

The value of AI in this space will depend on whether it can reduce uncertainty before expensive synthesis, formulation, and in vivo testing. For established ASO and siRNA chemistries, this may mean prioritizing modification patterns with lower predicted off-target or toxicity risk. For emerging chemistries, the more useful role may be different: identifying where data are too sparse to support confident prediction and directing experiments toward the positions, motifs, or modalities most likely to clarify structure–activity relationships. In that sense, AI-assisted design should be viewed less as an automated replacement for chemical reasoning and more as a tool for making the next experiment more informative.

### 6.2. Module-Level Validation for Hybrid and Emerging Architectures

Hybrid and emerging oligonucleotide architectures will require more rigorous validation than conventional modification patterns because their therapeutic function is distributed across multiple chemical or structural modules. In an aptamer–siRNA conjugate, the aptamer is expected to provide target-cell recognition, while the siRNA must remain competent for intracellular silencing. In a peptide-conjugated PMO, the peptide is expected to improve delivery or tissue access, while the PMO must retain splice-switching activity. In a cyclic or stimulus-responsive oligonucleotide, the inactive architecture must remain stable until the intended trigger restores target engagement. In a DNAzyme, XNAzyme, or aptamer-guided nanostructure, recognition, folding, catalytic activity, delivery, and release may all depend on different parts of the construct. These systems cannot be evaluated only by final potency because final activity does not reveal whether the targeting module, delivery module, activation mechanism, or effector strand performed as intended.

Future studies should therefore separate module performance from integrated construct performance. A hybrid oligonucleotide may appear effective because one module works extremely well, because another module compensates for a weakness, or because an unintended interaction dominates the biological outcome. For example, improved uptake does not necessarily mean productive intracellular localization; stronger target binding does not ensure catalytic turnover or splice correction; and conditional activation does not prove that release occurs at the intended site. This distinction is especially important for architectures that combine chemically modified strands with ligands, peptides, nanoparticles, cyclic constraints, or supramolecular assemblies. Each added component should have a defined purpose, and future studies should test whether that purpose remains measurable after the construct is assembled, delivered, and exposed to biological conditions.

This need for module-level validation also affects how emerging architectures should be compared with established platforms. A hybrid system should not be judged only against an unmodified oligonucleotide if the clinically relevant benchmark is a chemically optimized ASO, PMO, or siRNA. Similarly, a DNAzyme, XNAzyme, aptamer conjugate, or cyclic ASO should not be advanced solely because it improves stability or cellular uptake in isolation. The more important question is whether the added module improves the specific limitation that prevented the simpler architecture from being therapeutically useful. In this sense, hybrid design should be treated less as a way to add functionality and more as a testable hypothesis about which barrier limits the modality.

The future direction is to make hybrid architectures more mechanistically interpretable. Studies should be designed so that loss or modification of each module reveals its contribution to targeting, delivery, activation, target engagement, catalytic turnover, or therapeutic output. This would make it easier to distinguish architectures that create genuine functional gains from those that add chemical complexity without clarifying the source of activity. For emerging oligonucleotide therapeutics, the most useful hybrid designs will be those in which every added module has a measurable role and the integrated construct performs better because those roles remain coordinated in vivo.

### 6.3. Standardizing Translational Evidence for Modified Oligonucleotides

There is a need for more standardized reporting that links chemical modification choices to translational outcomes. Studies routinely report results related to potency, stability, uptake, or binding, but rarely the chemical detail needed to compare strategies across modalities. This would include not only the sequence but the placement of backbone, sugar, base, ligand, and scaffold-level features, together with stereochemistry, positional patterning, conjugation site, and activation state. Equally important is specifying what each modification is meant to do, such as improve delivery, resist degradation, alter protein interactions, control immune recognition, prolong duration, or regulate activation. Increased uptake matters little if a splice-switching oligonucleotide never reaches the nucleus; tighter binding can be counterproductive if a catalytic oligonucleotide cannot release its product; and longer persistence may be harmful if off-target interactions are prolonged in step. Without this level of detail, an observed benefit may not be confidently attributed to the modification rather than to sequence context, delivery method, or assay model. Standardized reporting expectations would make these relationships explicit and individual studies cumulative. For conventional ASO and siRNA platforms, this means clearer reporting of positional and stereochemical detail, tissue exposure, off-target transcript effects, and repeated-dose safety; for hybrid or catalytic systems such as aptamer conjugates, DNAzymes, XNAzymes, and oligonucleotide nanostructures, it means defining each functional module, confirming assembly or activation state, and demonstrating that the intended mechanism still operates after delivery, alongside how the construct was purified, characterized, and evaluated under biologically relevant conditions. Such standards would not replace empirical optimization, but they would better allow for distinguishing between structure–activity relationships from a system-specific observations. This is an important prerequisite for turning isolated modification studies into predictive design rules as these chemistries grow more complex.

## 7. Conclusions

Chemical modification has transformed oligonucleotides into a clinically validated therapeutic class, but the field has moved beyond the simple idea that greater stability, affinity, or nuclease resistance is inherently beneficial. The examples discussed in this review show that each modification changes more than one property at a time. A phosphorothioate linkage can improve persistence while altering protein interactions; a high-affinity sugar can increase potency while amplifying off-target hybridization; a scaffold replacement can support durable steric blockade while shifting the burden toward delivery; and a hybrid architecture can add targeting or activation while making the source of activity harder to interpret. These trade-offs are not exceptions to oligonucleotide design. They are the central design problem. The field has therefore moved beyond evaluating single modifications in isolation. Current oligonucleotide therapeutics increasingly rely on combined chemical strategies in which backbone, sugar, scaffold, and conjugation-based modifications are integrated within a single construct. This shift reflects the fact that no single modification fully addresses the major barriers facing oligonucleotide therapeutics, particularly with respect to delivery, intracellular access, durability, safety, and mechanism-specific activity. It also suggests that future advances will depend less on identifying isolated new chemistries and more on understanding how existing and emerging modifications can be combined to produce predictable, interpretable, and modality-specific therapeutic performance.

This function-first view is especially important because the same modification can be enabling, neutral, or disruptive depending on the modality in which it is placed. RNase H-active ASOs require architectures that preserve a cleavage-compatible DNA-like region. Splice-switching and steric-blocking oligonucleotides require durable occupancy in the correct compartment. RNAi therapeutics require chemically stabilized duplexes that remain compatible with Argonaute-mediated silencing and tissue delivery. Aptamers, decoys, CpG oligodeoxynucleotides, DNAzymes, ribozymes, and XNAzymes require preservation of folding, recognition, immune-receptor engagement, catalytic geometry, turnover, or product release. Chemical modification should therefore be evaluated not by whether it improves a single property but by whether it preserves the molecular event that makes the modality therapeutic. Clinical experience reinforces this function-first view. Approved oligonucleotide therapeutics have not sampled the full chemical space evenly; they cluster around chemistries and architectures that solve specific translational problems, such as systemic exposure for PS/2′-modified ASOs, durable steric blockade for PMOs, and hepatocyte delivery for stabilized siRNAs. Emerging chemistries such as FANA, tcDNA, MsPA, PN-type linkages, stereodefined PS designs, cyclic oligonucleotides, aptamer-guided conjugates, DNAzyme/XNAzyme systems, and nucleic-acid nanostructures expand the design space, but their clinical value will depend on whether they can link added chemical function to measurable improvements in delivery, safety, durability, or mechanism-specific activity. Experimental novelty alone is not enough; the added chemistry must remain interpretable under biological and clinical constraints.

Future progress will depend on moving from modification catalogs toward predictive, modality-aware design. This will require better chemistry-resolved datasets, standardized reporting of modification placement and architecture, module-level validation for hybrid systems, and computational tools capable of connecting sequence, chemistry, structure, delivery, and mechanism-specific outcomes. The central question for chemically engineered oligonucleotide therapeutics is no longer whether a modification can make a strand more stable or more potent in isolation. It is whether the modification helps the therapeutic molecule perform the right molecular task, in the right biological context, with an acceptable safety and translational profile. That framework offers a more critical and clinically relevant basis for designing the next generation of oligonucleotide therapeutics.

## Figures and Tables

**Table 1:** Shared and mechanism-specific design constraints across therapeutic oligonucleotide modalities.

Therapeutic Modality	Primary Mechanism	Mechanism-Specific Design Constraint
RNase H-dependent ASOs/gapmers	Bind target RNA and recruit RNase H-mediated RNA cleavage	Must preserve an RNase H-compatible DNA/RNA duplex region while maintaining target-site accessibility and limiting off-target hybridization.
Steric-blocking and splice-switching oligonucleotides	Bind RNA to alter splicing, block translation, or interfere with RNA–protein interactions without intentionally promoting target RNA cleavage	Require accessible RNA target sites, sustained occupancy, and, for splice modulation, effective nuclear access.
Micro RNA Mimics & Inhibitor	Restore, enhance, or inhibit miRNA-mediated regulation of target transcripts	Must account for network-level regulation, partial complementarity can affect multiple transcripts and may perturb endogenous miRNA pathways.
siRNAs/RNAi therapeutics	Use guide-strand loading into RISC to direct RNA silencing	Must preserve RNAi machinery compatibility, guide/passenger strand behavior, and minimize seed- or passenger-strand-mediated off-target effects.
Aptamers	Fold into defined structures that bind proteins, small molecules, or cellular targets	Must preserve secondary/tertiary structure and binding-site geometry while improving stability and persistence in biological environments.
CpG Deoxyoligonucleotides	Engage innate immune receptors, commonly TLR9, to stimulate or modulate immune signaling	Must balance nuclease resistance with controlled receptor engagement while avoiding excessive inflammatory activation.
DNAzymes and ribozymes	Use sequence-guided catalytic nucleic acid activity to promote target-site reactions, commonly RNA cleavage or RNA-processing reactions in gene-regulation contexts	Must preserve substrate recognition, catalytic-core integrity, folding, and, where relevant, metal ion or cofactor compatibility.
Decoy oligonucleotides	Mimic regulatory DNA elements and sequester transcription factors or other DNA-binding proteins	Must preserve protein-binding recognition while improving stability, intracellular persistence, and nuclear availability.

**Table 2:** Phosphate-linkage modifications and scaffold-replacement classes.

Modification	Description	Pros	Cons
**Phosphate-linkage modifications—native sugar–phosphate framework retained; combinable with one another and with sugar modifications**			
Phosphorothioate (PS)	Non-bridging phosphate oxygen replaced by sulfur; backbone stays anionic; chiral phosphorus center (Rp/Sp). PS-DNA remains an RNase H substrate.	Improves nuclease resistance and plasma-protein binding/tissue exposure; compatible with gapmer DNA gaps; terminal protection in siRNA duplexes.	Nonspecific protein binding can drive off-target/toxicity effects; stereochemical heterogeneity unless stereodefined; small per-linkage affinity penalty.
Mesyl phosphoramidate (MsPA/μ)	Non-bridging oxygen replaced by an N-methanesulfonyl amido group; backbone remains anionic and a close phosphodiester mimic. Recruits RNase H with a DNA sugar.	Higher RNA affinity, nuclease stability, and specificity than PS; lower protein-binding toxicity (lacks the sulfur pharmacophore).	Less clinically validated; for splice-switching must be paired with a 2′-modified sugar to abort RNase H.
Phosphoryl guanidine (PN)	Non-bridging oxygen replaced by a guanidino group; largely charge-neutral. RNase H compatibility is design-dependent.	Tunable charge, pharmacokinetics, and durability; reduced charge-dependent interactions.	Narrow validation across modalities; neutral charge can perturb uptake or folding in structure-dependent systems.
Alkylphosphonate (e.g., methylphosphonate)	Non-bridging oxygen replaced by an alkyl group; neutral/reduced charge; chiral phosphorus center.	Strategic placement reduces charge-dependent toxicity and seed-mediated off-targeting.	Full substitution reduces solubility/activity; best used as a positional modifier rather than a complete replacement.
Boranophosphate	Non-bridging oxygen replaced by a borane (BH_3_) group; backbone stays anionic; chiral phosphorus center.	Nuclease resistance while retaining RNAi activity in selected scaffolds.	Synthetic complexity; less clinically validated.
**Scaffold-replacement classes—entire backbone replaced; neutral; not combinable with native-backbone chemistries**			
Phosphorodiamidate morpholino oligomer (PMO)	Backbone replaced by morpholine rings (saturated heterocycles) joined by neutral phosphorodiamidate linkages; neutral. Not an RNase H substrate.	High nuclease resistance; low immunogenicity; durable steric blockade; clinically validated (DMD exon skipping).	Neutral scaffold limits charge-dependent uptake; delivery and nuclear access are the main limitations.
Peptide nucleic acid (PNA)	Sugar–phosphate backbone replaced by a neutral N-(2-aminoethyl)glycine pseudopeptide scaffold; neutral. Not an RNase H substrate.	Very high sequence-selective affinity; resistant to nucleases and proteases; strand invasion in some DNA contexts.	Poor solubility/aggregation; inefficient cellular uptake; high affinity can slow target dissociation.

**Table 3:** Sugar and conformational modifications.

Modification	Description	Pros	Cons
**Flexible 2′-substituted ribose analogues**			
2′-O-methyl (2′-OMe)	2′-OH replaced by 2′-O-methyl; shifts sugar toward C3′-endo. Does not support RNase H.	Improves affinity; reduces immunostimulation; widely used in siRNA and ASO wings.	Only modest nuclease resistance on its own—typically combined with PS or bulkier modifications.
2′-O-methoxyethyl (2′-MOE)	2′-OH replaced by 2′-O-methoxyethyl. Does not support RNase H.	Strong gains in duplex stability, affinity, and nuclease resistance; clinically validated (nusinersen, inotersen).	Bulky substituent; wing-only in gapmers.
2′-Fluoro (2′-F)	2′-OH replaced by fluorine in the ribo configuration; strongly favors C3′-endo/A-form. Does not support RNase H.	High affinity and thermal stability; heavily used in siRNA.	Modest nuclease resistance alone (usually paired with PS); context-dependent safety considerations.
**Arabino-configured analogue**			
2′-Fluoro-arabino nucleic acid (FANA)	2′-fluoro in the arabino configuration; behaves as a DNA mimic. Supports RNase H (FANA:RNA hybrids activate RNase H1).	Combines nuclease resistance, high affinity, and RNase H recruitment; compatible with ASO, siRNA, aptamer, and XNAzyme contexts.	Less clinically established than 2′-O-alkyl or LNA chemistries; still emerging.
**Conformationally constrained sugars**			
Locked nucleic acid (LNA)	Methylene bridge links 2′-O to 4′-C, locking a rigid C3′-endo conformation. Does not itself support RNase H.	Large Tm/affinity gains; potent in short gapmer wings.	High affinity can stabilize off-target pairing; associated with sequence-dependent, RNase H1-mediated hepatotoxicity in gapmers.
Constrained ethyl (cEt)	2′,4′-constrained 2′-O-ethyl bridge; locked like LNA but with improved tolerability. Does not support RNase H.	LNA-like high affinity with a generally improved therapeutic index in gapmer wings.	Still high-affinity, so off-target risk remains; performance is placement-sensitive.
Tricyclo-DNA (tcDNA)	Conformationally constrained DNA analogue with additional fused carbocyclic rings. Fully modified tcDNA does not support RNase H.	High affinity; favorable tissue distribution (muscle, CNS) in neuromuscular models; durable steric occupancy.	Developing scaffold, not yet clinically established; fully modified form is unsuited to RNase H mechanisms.

**Table 4:** Chemistry, delivery, and status of FDA-approved oligonucleotide therapeutics.

Drug (Trade Name)	Target/Mechanism	Core Chemistry	Delivery/Route	Status
**RNase H-dependent antisense oligonucleotides**				
Fomivirsen (Vitravene)	CMV IE2 mRNA	21-mer; 20 PS, unmodified deoxyribose	Intravitreal	Discontinued (withdrawn 2006)
Mipomersen (Kynamro)	ApoB-100 mRNA	20-mer 5-10-5 gapmer; 19 PS, 10 2′-MOE, 9 5-Me-C	Subcutaneous	Discontinued (hepatotoxicity, injection-site reactions)
Inotersen (Tegsedi)	TTR mRNA	20-mer 5-10-5 gapmer; 19 PS, 10 2′-MOE, 5 5-Me-C	Subcutaneous	Discontinued
Tofersen (Qalsody)	SOD1 mRNA	20-mer 5-10-5 gapmer; 15 PS + 4 PO, 10 2′-MOE, 5 5-Me-C	Intrathecal	Approved 2023
Eplontersen (Wainua)	TTR mRNA	20-mer 5-10-5 gapmer; 13 PS, 10 2′-MOE, 5 5-Me-C, GalNAc	Subcutaneous	Approved 2023
Olezarsen (Tryngolza)	apoC-III mRNA	20-mer 5-10-5 gapmer; 19 PS, 10 2′-MOE, 5 5-Me-C, GalNAc	Subcutaneous	Approved 2024
Donidalorsen	Prekallikrein mRNA	GalNAc-conjugated gapmer	Subcutaneous	Approved 2025
**Steric-blocking and splice-switching oligonucleotides**				
Nusinersen (Spinraza)	SMN2 pre-mRNA (ISS-N1)	18-mer; 17 PS, fully 2′-MOE, 5-Me-C	Intrathecal	Approved 2016
Eteplirsen (Exondys 51)	Dystrophin exon 51	30-mer; 30 phosphorodiamidate, 30 morpholino	Intravenous	Approved 2016
Golodirsen (Vyondys 53)	Dystrophin exon 53	25-mer; 25 phosphorodiamidate, 25 morpholino	Intravenous	Approved 2019
Viltolarsen (Viltepso)	Dystrophin exon 53	21-mer; 20 phosphorodiamidate, 21 morpholino	Intravenous	Approved 2020
Casimersen (Amondys 45)	Dystrophin exon 45	22-mer; 22 phosphorodiamidate, 22 morpholino	Intravenous	Approved 2021
**RNA interference therapeutics**				
Patisiran (Onpattro)	TTR mRNA	21 + 21-mer; 9/2 2′-OMe only; no PS, no 2′-F	Lipid nanoparticle, IV	Approved 2018
Givosiran (Givlaari)	ALAS1 mRNA	21 + 23-mer; sense 2 PS, 16 2′-OMe, 5 2′-F, GalNAc; antisense 4 PS, 12 2′-OMe, 11 2′-F	GalNAc, subcutaneous	Approved 2019
Lumasiran (Oxlumo)	HAO1 mRNA	21 + 23-mer; sense 2 PS, 17 2′-OMe, 4 2′-F, GalNAc; antisense 4 PS, 17 2′-OMe, 6 2′-F	GalNAc, subcutaneous	Approved 2020
Inclisiran (Leqvio)	PCSK9 mRNA	21 + 23-mer; sense 2 PS, 18 2′-OMe, 2 2′-F, GalNAc; antisense 4 PS, 13 2′-OMe, 10 2′-F	GalNAc, subcutaneous	Approved 2021 (EU)/2022 (FDA)
Vutrisiran (Amvuttra)	TTR mRNA	21 + 23-mer; sense 2 PS, 17 2′-OMe, 4 2′-F, GalNAc; antisense 4 PS, 18 2′-OMe, 5 2′-F	Triantennary GalNAc, subcutaneous	Approved 2022
Nedosiran (Rivfloza)	LDHA mRNA	36 + 22-mer; sense 1 PS, 23 2′-OMe, 9 2′-F, 4 GalNAc; antisense 5 PS, 12 2′-OMe, 10 2′-F	GalNAc, subcutaneous	Approved 2023
Fitusiran (Qfitlia)	Antithrombin mRNA	21 + 23-mer; sense 2 PS, 9 2′-OMe, 12 2′-F, GalNAc; antisense 4 PS, 14 2′-OMe, 9 2′-F	Triantennary GalNAc (L96), subcutaneous	Approved 2025
Plozasiran	apoC-III mRNA	GalNAc-conjugated siRNA	ESC-GalNAc, subcutaneous	Approved 2025
**Aptamers**				
Pegaptanib (Macugen)	VEGF165 protein	28-mer; PEG, 12 2′-OMe, 13 2′-F	Intravitreal	Discontinued
Avacincaptad pegol (Izervay)	Complement C5 protein	39-mer; PEG, 14 2′-OMe, 21 2′-F	Intravitreal	Approved 2023
**Other**				
Imetelstat (Rytelo)	Telomerase RNA template	13-mer; 13 PS, 13 3′-amino (N3′→P5′ phosphoramidate), fatty-acid conjugate	Intravenous	Approved 2024
Defibrotide (Defitelio)	Mechanism not fully elucidated	Polydisperse mixture (~4 to ~100-mer); predominantly single-stranded polydeoxyribonucleotide, porcine intestinal origin; unmodified	Intravenous	Approved

Lengths, modification counts, and discontinuation status from FDA/CDER (Division of Product Quality Assessment; listing current as of 30 April 2025); donidalorsen and plozasiran post-date that listing. PS = phosphorothioate; PO = phosphodiester; 2′-OMe = 2′-O-methyl; 2′-F = 2′-fluoro; 2′-MOE = 2′-O-methoxyethyl; 5-Me-C = 5-methylcytosine; GalNAc = N-acetylgalactosamine.

## Data Availability

No new data were created or analyzed in this study. Data sharing is not applicable.

## Abbreviations

The following abbreviations are used in this manuscript:

2′-F	2′-Fluoro
2′-MOE	2′-O-Methoxyethyl
2′-OMe	2′-O-Methyl
ASO	Antisense oligonucleotide
C5	Complement factor 5
CpG ODN	Cytosine–phosphate–guanine oligodeoxynucleotide
DNAzyme	Deoxyribozyme
FANA	2′-Fluoro-arabino nucleic acid
FBS	Fetal bovine serum
GalNAc	N-Acetylgalactosamine
LNA	Locked nucleic acid
miRNA	MicroRNA
mRNA	Messenger RNA
ODN	Oligodeoxynucleotide
ONT	Oligonucleotide therapeutic
PNA	Peptide nucleic acid
PMO	Phosphorodiamidate morpholino oligomer
PS	Phosphorothioate
RISC	RNA-induced silencing complex
RNAi	RNA interference
RNase H	Ribonuclease H
SELEX	Systematic Evolution of Ligands by Exponential Enrichment
siRNA	Small interfering RNA
SSO	Splice-switching oligonucleotide
TLR9	Toll-like receptor 9
Tm	Melting temperature
TTR	Transthyretin
UTR	Untranslated region
XNAzyme	Xeno nucleic acid enzyme
